# Variability in pediatric and neonatal organ offering, acceptance and utilization: a survey of Canadian pediatric transplant programs and organ donation organizations

**DOI:** 10.3389/frtra.2024.1458563

**Published:** 2024-09-27

**Authors:** Laurie A. Lee, Augustina Okpere, Dori-Ann Martin, Meagan Mahoney, Lee James, Yaron Avitzur, Bailey Piggott, Christopher Tomlinson, Simon Urschel, Lorraine Hamiwka

**Affiliations:** ^1^ Faculty of Nursing, University of Calgary, Calgary, AB, Canada; ^2^ Department of Pediatrics, Cuming School of Medicine, University of Calgary, Calgary, AB, Canada; ^3^ Alberta Children’s Hospital Research Institute, University of Calgary, Calgary, AB, Canada; ^4^ Department of Pediatric Critical Care, Alberta Health Services, Calgary, AB, Canada; ^5^ Canadian Blood Services, Vancouver, BC, Canada; ^6^ Division of Gastroenterology, Hepatology and Nutrition, SickKids Hospital, Toronto, ON, Canada; ^7^ Department of Paediatrics, University of Toronto, Toronto, ON, Canada; ^8^ Neonatal Intensive Care, Hospital for Sick Children, Toronto, ON, Canada; ^9^ Pediatric Cardiology, Stollery Children’s Hospital, Edmonton, AB, Canada; ^10^ Faculty of Medicine and Dentistry, University of Alberta, Edmonton, AB, Canada

**Keywords:** organ donation organizations, pediatric transplant programs, organ donation, transplantation, transplant policies, pediatric recipients, paediatrics

## Abstract

**Introduction:**

Solid organ transplantation in children is a lifesaving therapy, however, pediatric organ donation rates remain suboptimal.

**Methods:**

We conducted a cross-sectional survey of Canadian organ donation organizations (ODOs) and pediatric transplant programs (TPs), aiming to describe policies and practices for pediatric organ allocation, acceptance, and utilization in Canada.

**Results:**

Response rates were 82% and 83% respectively for ODOs and transplant programs comprising 7 kidney, 3 heart, 2 lung, 2 liver and 1 intestine programs. All 9 ODOs reported offering pediatric organs following death by neurological criteria (DNC), while 8 reported offering organs following death by circulatory criteria (DCC) for some organs. Variability was found across ODOs and TPs. There was little agreement on both absolute and organ-specific donor exclusion criteria between ODOs. There was further disagreement in organ specific acceptance criteria between ODOs and TPs and between TPs themselves. Notably, despite the development of pediatric donation after DCC guidelines, organs from DCC donors are excluded by many ODOs and TPs.

**Discussion:**

Further variability in pediatric specific training, policies, and allocation guidelines are also documented. Significant areas for improvement in standardization in organ acceptance, offering, and allocation in pediatric donation and transplantation across Canada were identified.

## Introduction

Organ transplantation is the standard treatment for most pediatric patients with end stage organ failure with the advantage of improving survival and quality of life (1, 2). Although, there has been an increase in organ donation in adults, the disparity between organ demand and supply persists (3). In the United States of America (USA), despite polices that prioritize deceased organ allocation to children, pediatric deceased donor organ engraftment constitutes approximately 6.5% of all transplants and many children still die on, or are withdrawn from transplant waitlists for deterioration (3–6). In Canada, pediatric donation rates remained unchanged in contrast to increasing adult donation rates and despite the publication of pediatric guidelines for donation after death by circulatory criteria (DCC) in 2017 (3). Factors contributing to low rates of pediatric donation and transplantation include low pediatric mortality rates, low referral rates (7), perceived challenges discussing donation with bereaved parents (8), lack of experience with pediatric DCC donation (9) and the complexity of organ acceptance and allocation guidelines for pediatric donors and recipients (10, 11).

Globally, there is evidence of wide variability in policies and practices within pediatric intensive care unit (PICU) and neonatal intensive care unit (NICU) donation programs, organ donation organizations (ODOs) and transplant programs (TPs) which likely contribute to missed donation and transplant opportunities (12–18).

In Canada, organ allocation, acceptance and utilization are guided by local, provincial, and national policies which differentially impact practices across provinces. A survey of adult kidney TPs across Canada demonstrated wide variability in practice across the TPs and a high rate of non-acceptance of donor kidneys (19). While understanding organ acceptance practices within individual transplant programs is important, organ offering, acceptance and utilization is impacted by external organizations including ODOs. Furthermore, in pediatric transplantation, given the scarcity of pediatric organs, and the significant sharing of organs provincially and nationally, understanding of the practices of pediatric TPs and ODOs in a national context is essential to identify inconsistencies and eliminate barriers. This study aimed to describe the current policies and practices of pediatric ODOs and TPs in pediatric organ acceptance and utilization, and to identify facilitators and barriers to pediatric organ donation and transplantation within ODOs and TPs.

## Materials and methods

### Study design and ethical considerations

A self-administered, web-based cross-sectional survey was administered to all Canadian ODOs and pediatric TPs. The study was approved by the Conjoint Health Research Ethics Board at the University of Calgary (REB 21-0021). A letter of information preceded the survey and participation constituted implied consent to collect and publish data. The survey is reported in accordance with the Checklist for Reporting of Survey Studies (CROSS) guidelines (20) (Supplementary File 1).

### Survey development

The details of the survey development are reported elsewhere (21). The survey was developed with key parties from pediatric and neonatal organ donation and transplant communities using the methodology described by Burns et al. (22). The questionnaires (Supplementary Files 2, 3), written in French and English, were pre-tested for readability, flow, clinical sensibility, and construct validity among all members of the survey development team and one external donation and pediatric critical care expert. The term pediatric was used inclusively for all potential donors and recipients aged 0–18 years of age, inclusive of eligible neonates.

### Study setting, sample, and administration

The survey was administered to the administrative leads of ODOs covering pediatric organ donation in Canada and the medical and/or surgical lead/delegate for each pediatric TPS within Canada via Interceptum® between January 2021 and April 2022. The leads of ODOs and TPs were identified through established networks in Canadian Blood Services, a National Donation Network, and the Pediatric Group executive database of the Canadian Society of Transplantation. Email invitations with a standardized letter containing study information were sent to the lead of each TP/ODO. For TPs reminder emails were sent after 2 weeks, 1 month, with one further follow-up via telephone or additional email after 3 months. For ODOs reminder emails were sent at 3, 6 and 9 weeks. Leads were encouraged to discuss the survey with their teams to ensure that the responses were representative of the practices of their corresponding ODOs/TP. Each ODO/TP completed one survey.

### Data analysis

Survey results were cleaned and exported into Microsoft Excel (Microsoft Corporation). Categorical data were summarized using descriptive statistics. Open ended questions were inductively coded utilizing content analysis (23, 24).

## Results

**Figure 1:**
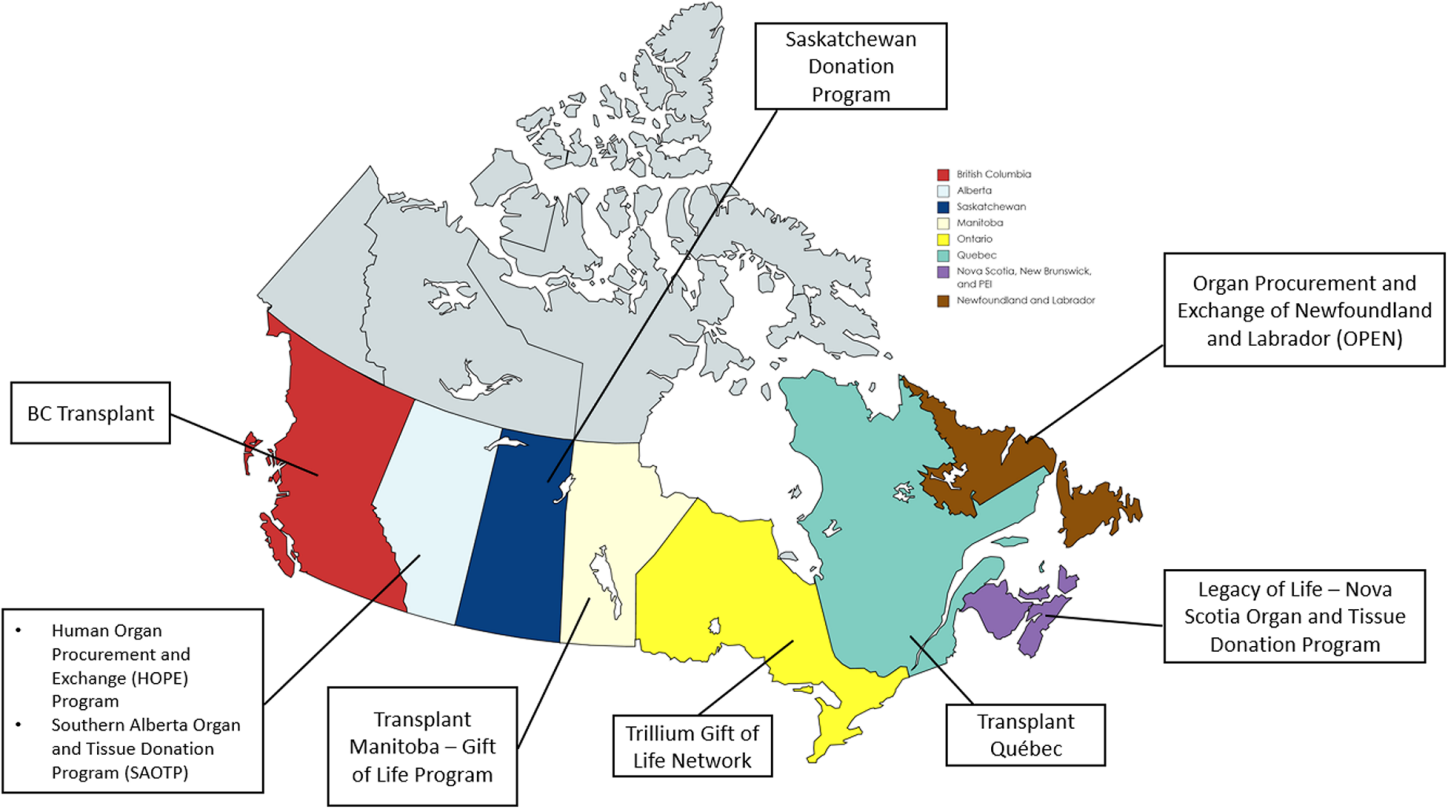
Participating organ donation organizations (ODOs) across Canada. Created with map-chat.net.

Of the 11 ODOs approached, 9 (82%) completed surveys. This represents the ODOs covering all pediatric/neonatal organ donation and transplantation in Canada (Figure 1). Two ODOs did not complete the survey because they refer all pediatric cases to an ODO in another province. Of the 18 pediatric TPs approached, 15 (83%) completed the survey. These included 7 kidney, 3 heart, 2 lung, 2 liver and 1 intestine TPs. The fewest number of transplanted organs per program were lungs and intestines, with an estimated 1–5 transplants per annum, while the largest number of transplants per program occurred in one kidney and one liver program with each indicating >20 transplants per annum. Deceased donor organ offers were reviewed by only the transplant physician in 7 programs, both the transplant physician and surgeon in 5 programs, and solely the transplant surgeon in 3 programs.

### Characteristics of organ transplant recipients

There was variation in the ages of pediatric organ recipients across the programs. Six (86%) kidney programs reported transplanting children ≥2 years while 1 program transplanted children from 18 months of age. All liver, heart, and 1/2 lung programs reported no minimum age for transplant, with the other lung program indicating a minimum age of 3 years. The single intestine program transplanted children from 6 months of age.

### Absolute donor exclusion criteria by ODOs

Three ODOs did not report any absolute pediatric donor exclusion criteria. One ODO listed an absolute donor exclusion age of less than 1 year. Donor weight was cited as an absolute exclusion by one ODO for donors <2.2 kg and by another ODO for donors <8 kg, for potential recipients within the same province. Two ODOs cited infants born at <36 weeks gestation as an absolute donor exclusion and 3 ODOs reported not accepting potential donors with hepatitis B, hepatitis C and human immunodeficiency virus infection.

### Absolute organ specific criteria for acceptance, offering and utilization by ODOs and TPs

Absolute exclusion criteria for specific organs from pediatric donors varied between ODOs and TPs (Tables 1, 2).

**Table 1:** Exclusion criteria for pediatric organs reported by Canadian organ donation organizations.

Organ	Age based exclusion criteria	Weight	Exclusion criteria
Heart (*n*^a^ = 2)	<1 year (DCC heart) <36 weeks (DCC)	<2 kg (DCC)	DCC donor
Lung (*n* = 1)	<10 years	<20 kg	DCC donor
Kidney (*n* = 6)	<3 months <1 year	<8 kg offered nationally <2.5 kg <5 kg	Chronic renal failure Peak Cr >300
Liver (*n* = 1)	<1 month (DCC) <36 weeks	<2 kg (DCC)	DCC donor
Intestine (*n* = 1)	<36 weeks		DCC donor
Pancreas/Islet Cells (*n* = 1)	<10 years (Islet cells)		DCC donor

^a^ *n* denotes the number of ODOs that reported an exclusion criteria for each organ.

**Table 2:** Absolute donor exclusion criteria reported by pediatric transplant programs in Canada.

Organ (number of programs)	Donor age	Donor weight	DCC donor	HLA matching	Presence of high level class I/II HLA antibodies	Organ function	Expected cold ischemic time	Other
Kidney (*n* = 7)^a^	• <5 or >45 years • >50 years excluded • <7–8 years • KDPI instead of age • >45 years	• 20 kg recipients are screened for “small donors” • <20kg No exclusion <12 kg	• Excluded (*n* = 2) • Only if KDPI <35 • Excluded for regular urgency	• Minimum 1 class II match • Minimum 1–2 class II match • Minimum1B 1DR (*n* = 2)	• Not accepted	• Donor Cr >150, proteinuria (*n* = 2) • Donor AKI • CK >5,000–10,000 and increasing • GFR >90–100	• >18 h • >12 h • >18–24 h (high urgency <24 h) • >24 h on inotropes	NR
Heart (*n* = 3)	• >40 years • >50 years	• <70% or >300% of recipient weight • <80% or >300% recipient weight (discussion of outside if high urgency recipient) • <80% or >250% recipient weight	• Non-Local DCC (WIT 30–60 min) • DCC (*n* = 2)	• Virtual crossmatch positive for regular urgency, pre-existing DSA considered allowable for high urgency • HLA matched when appropriate but will transplant highly sensitized patients	• Exclusion for regular, considered for high urgency (*n* = 2)	• Ejection Fraction <40% • Ejection Fraction <55% • Abnormal cardiac function labs	• >6 h	NR
Liver (*n* = 2)	• <2 months • >45 years	• 10:1 donor: recipient weight	• Excluded • Generally not (consider for urgent with WIT <30 min)	• No exclusion (*n* = 2)	• No exclusion (*n* = 2)	• High transaminases and poor liver perfusion • Abnormal or worsening liver function	• >4 h (high urgency >8 h)	NR
Lung (*n* = 2)	• No exclusion (*n* = 2)	• >10% size/weight discrepancy; no more than 10 cm mismatch • Match according to predicted TLC	• No exclusion (*n* = 2)	• No exclusion (*n* = 2)	• Class II exclusion, except high urgency	• Exclude if O2 challenge gives PO2 <300, poor chest x-ray • Standard assessment, if poor function use Toronto *ex-vivo* lung perfusion technique to assess	• No exclusion	• Smoking history
Intestine (*n* = 1)	• <6 months or >55 years	• >150% of recipient weighs in select cases (ideal between 35 and 130%)	• Not accepted	• No absolute exclusion	NR	NR	• <4 h for regular urgency: <6 h for high urgency (ideal target <8 h)	NR

^a^ *n* denotes the number of transplant programs reporting, where no *n* reported, criteria only reported by one program.

#### Heart

One ODO reported excluding donors based on infection, while another reported excluding donation of DCC hearts from donors weighing <2 kg and neonates born at <36 weeks gestational age for neonatal recipients, and donors ≤1 year for pediatric recipients. There was also variability in the exclusion criteria across the three heart TPs. One reported exclusion of donors >40 years; another reported >50 years, and the third reported no absolute exclusion criterion. In all 3 programs, there was no consensus on donor's weight, cold ischemic time, or ejection fraction (range <40% and <50%). The only consistency across all 3 programs was willingness to accept high risk donors defined as high level class I and II HLA antibodies in high urgency cases. HLA mismatch was not considered an exclusion criterion. With regards to DCC organs, all three programs reported excluding adult DCC organs while 2 programs reported acceptance and utilization of pediatric DCC organs for status 4 patients on extracorporeal membrane oxygenation (ECMO) or other high intensity mechanical support with expected survival <2 weeks (Tables 1, 2).

#### Liver

Only one ODO reported absolute exclusion criteria for liver donation, excluding donors <1 month of chronological age for DCC donors and infants born at <36 weeks gestation and/or <2 kg, for all donors. Across liver TPs, one reported excluding donors <2 years while the other reported excluding donors >45years but would consider donors of all ages in some cases. Both TPs reported excluding adult DCC donors; however, one program would accept pediatric DCC donors in urgent cases. Neither TP reported excluding HLA mismatch (Tables 1, 2).

#### Lung

One ODO excluded lung donation from infants <2 kg and age <36 weeks gestation, and another excluded donation from children <10 years or <20 kg. In TPs, one program would consider age matching for donor and recipient, while the other would not accept donors when there was a greater than 10% size/weight discrepancy between the donor and the recipient. One TP reported utilizing donor's predicted total lung capacity rather than weight. HLA matching was not an exclusion criterion in one program. Both programs reported not having specific criteria for DCC donors but would reject adult DCC organs. Both programs reported willingness to accept class I HLA antibodies and pediatric DCC organs (Tables 1, 2).

#### Kidney

Absolute exclusion criteria for kidneys varied between ODOs with one citing age <3 months and another <1 year. Three ODOs cited 3 different weight restrictions as absolute exclusion criteria: <2.5 kg, <5 kg, and <8 kg. There was no agreement on age restrictions for the 4 kidney TPs who reported donor age as an exclusion criterion. Ages listed were <5 years and >45 years, >50 years, and use of kidney donation profile index (KDPI) <35% rather than donor's absolute age. While one TP reported accepting donors <20 kg for “small” patients, another reported excluding donors <20 kg. There was also wide variability in acceptance criteria of DCC organs. Two kidney TPs reported rejecting adult DCC donor organs, while 1 program reported they would accept in high urgency cases. Three programs reported accepting pediatric DCC kidneys. Of the programs that would accept DCC donors, the criteria varied, ranging from time to death <1 h; minimum of 2 HLA matches, cold ischemic time <12 h; KDPI <35%, warm ischemic time <30 min, cold ischemic time ranging 18–24 h across programs and low HLA mismatch and low risk of infections. The only consistent factors found were 2 kidney programs both reporting donor elevated serum creatinine and donor proteinuria for exclusion, and minimum of IB and 1DR human leucocyte antigen (HLA) matching for inclusion criteria (Tables 1, 2).

#### Intestine/islet cell

One ODO restricted bowel donation to infants born at >36 weeks gestation and reported excluding bowels from DCC donors. One ODO reported restricting islet cell donation to children >10 years of age. The only intestinal TP reported not accepting DCC organs for their patients (Tables 1, 2).

### Practices for offering specific organs by ODOs to TPs

**Figure 2:**
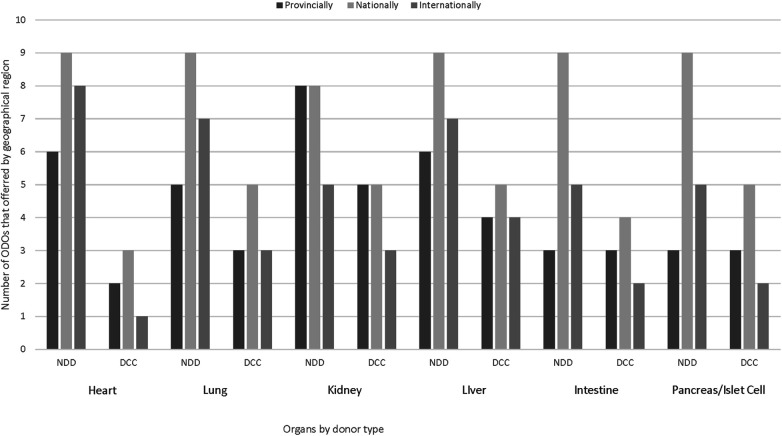
Geographic offering of pediatric/neonatal organs from DNC and DCC donors by Canadian ODOs. DNC, death by neurological criteria; DCC, death by circulatory criteria.

Practices for organ offering by ODOs to TPs varied by organ, ODO and location of recipient (provincial, national or international). The majority of ODOs offer organs from DNC donors nationally, with all 9 ODOs offering hearts, lungs, liver, intestine and pancreas/islet cells nationally and 8 offering kidneys nationally. For organs retrieved from DCC donors, 5 ODOs cited offering lungs, kidneys, livers, and pancreas/islet cells nationally, 4 reported offering intestines nationally and 3 offered hearts nationally. Provincial referral of organs, regardless of donor type, was limited by the lack of TPs within some provinces, but national and international offering still occurred from those ODOs (Figure 2).

**Figure 3:**
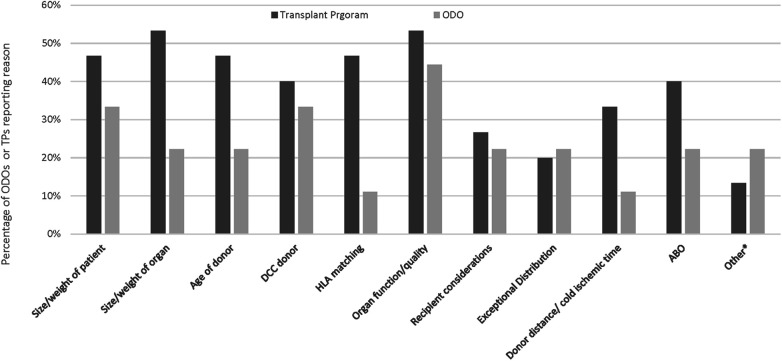
Reasons for ODOs not offering organs or TPs declining as reported by Canadian ODOs. Other reasons for TP decline included pandemic related and ODO not offering included lack of interest from the first several TPs, and donor instability.

The offering process for standard organs (i.e., not high risk/exceptional distribution) varied among ODOs; 4 cited offering organs only after consent had been obtained and workup completed; in 3 ODOs, organ offering depended on type of donor, time of day and workload. For non-standard organs, only 1 ODO reported waiting until consent and work up were completed before offering, while 3 ODOs reported offering organs/showing interest before approaching potential donor's families. ODOs use a variety of communication methods (e.g., fax, database system, phone) to offer organs, with most using 2 or more methods of communication (Figure 3).

### Organ utilization and non-utilization

**Figure 4:**
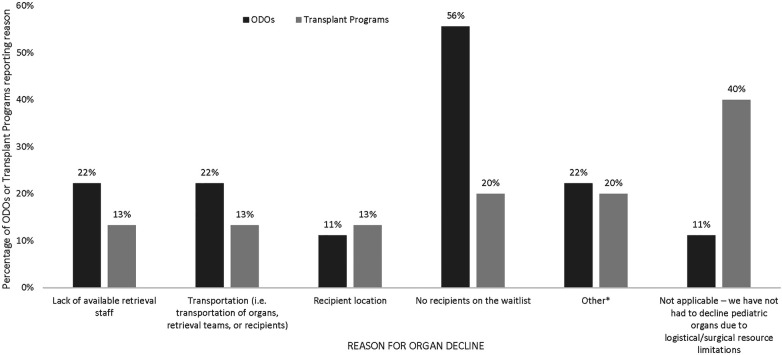
Reasons for ODOs not offering organs or TPs declining organs as reported by Canadian ODOs. Other reasons for TP decline included pandemic related and ODO not offering included lack of interest from the first several TPs, and donor instability.

The most common reasons reported by ODOs for ODO non-offering of organs was organ function quality and DCC donor (Figure 4). The most common reasons reported by ODOs for TP non acceptance of organs was size/weight of organ and organ function/quality (Figure 4). Other reasons for ODO non-offering and TP non-acceptance as reported by ODOs are reported in Figure 4.

**Figure 5:**
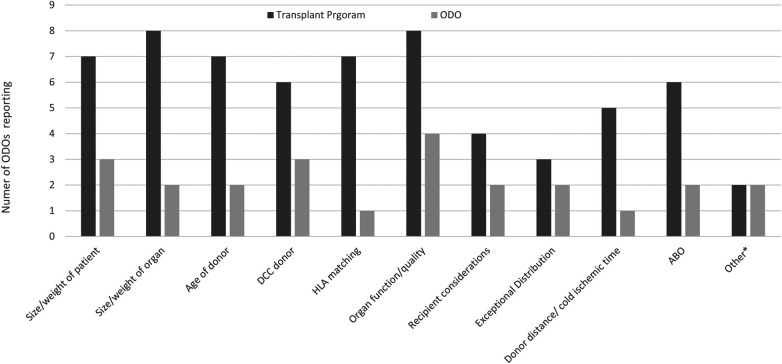
Logisitical reasons for pediatric organ decline as reported by Canadian ODOs and TPs in the past 5 years. Other reasons listed by ODOs were pandemic related, other reasons listed by TPs were adult TP program as first point of contact and pandemic related.

The majority of ODOs (8/9) and TPs (9/15) reported declining organs due to logistic difficulties (Figure 5). The most common logistical reason for organ decline was no recipients on the waitlist, reported by 5/9 ODOs and 3/15 TPs, and transportation issues reported by 2/9 ODOs and 3/15 TPs. Other logistical barriers are reported in Figure 5.

### Policies, procedures and education

#### ODO

The majority of ODOs (8/9) reported having specific pediatric policies, procedures and standardized pediatric donor management guidelines. Six identified a pediatric champion and only five ODOs reported providing pediatric specific education.

#### TP

**Figure 6:**
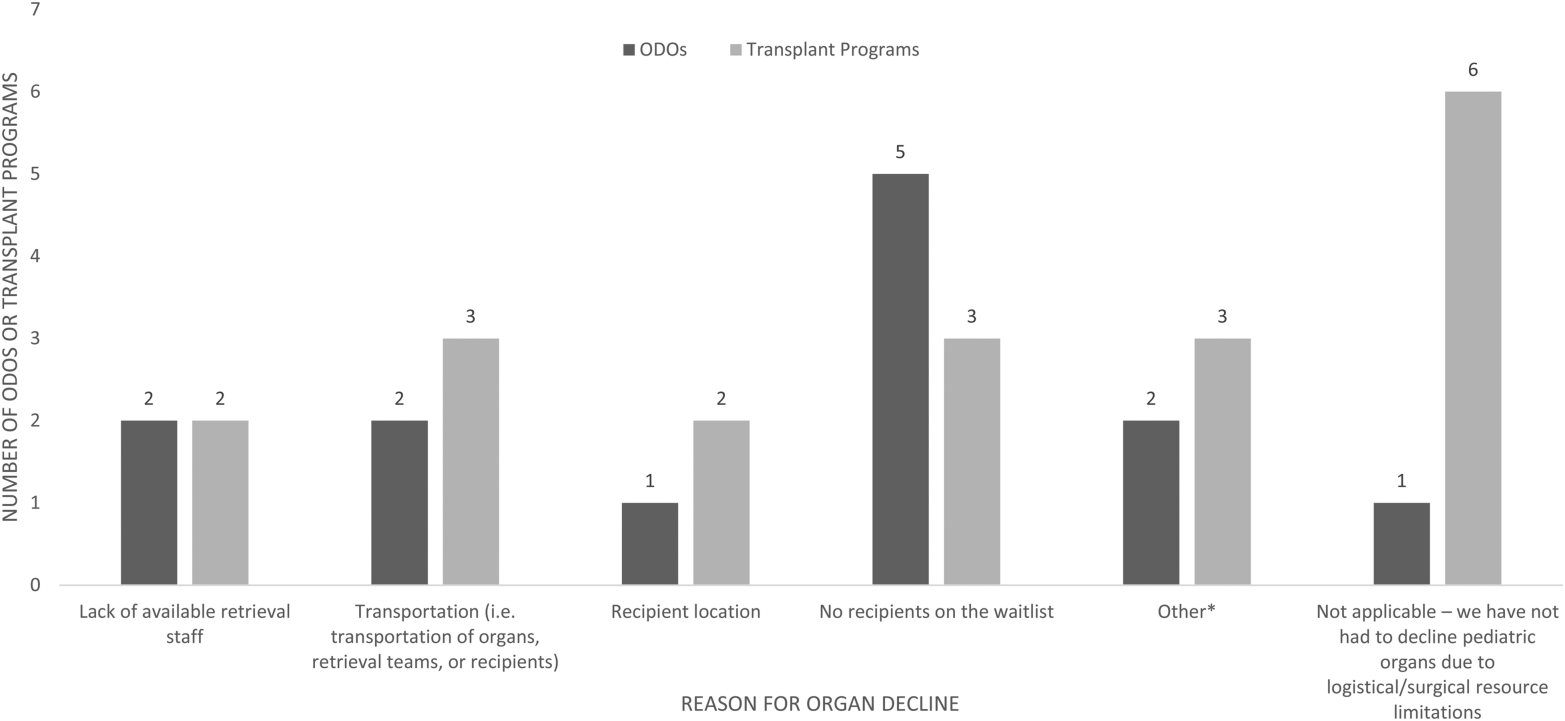
Logisitical reasons for pediatric organ decline as reported by Canadian ODOs and TPs in the past 5 years. Other reasons listed by ODOs were pandemic related, other reasons listed by TPs were adult TP program as first point of contact and pandemic related.

TPs reported being guided by local, provincial, national and international policies but this differed across TPs, even within the same organ (Figure 6). Overall, a majority (11/15) reported being guided by more than one policy (Figure 6). Across all organs 8/15 TPs reported having specific policies for pediatric organ allocation. Those that reported not having specific policies for pediatric organ allocation (7/15), reported being guided by either provincial TP guidelines, case by case discussion, priority for pediatric donors to pediatric recipients, or policies from other centres. The majority of TPs (14/15) reported the use pediatric specific recipient management guidelines.

### Registries and record keeping

#### ODO

All ODOs cited reporting data to local, national, or international registries and as well as maintaining their own database within their program. Four reported collecting donation and transplant data, another 4 cited collecting only donation data, and 1 only transplant data. While 7 ODOs reported collecting pediatric specific data, only 5 reported recording information on pediatric deaths or removal from transplant waitlists. All participating ODOs indicated that they record information on both pediatric/neonatal organs declined by TPs as well as the reasons for not offering pediatric/neonatal organs. Only 2 ODOs have pediatric specific committees and only one of these has family involvement. Four ODOs reported reviewing all pediatric deaths for donor potential; 3 reported reviewing pediatric but not neonatal deaths, and 2 reported not reviewing pediatric or neonatal deaths. Of the 7 ODOs that review pediatric/neonatal deaths, 5 reported these audits externally to the ODO.

#### TP

The majority of TPs (14/15) reported collecting and reporting data to local, national or international registries. Nine programs reported collecting data on patient removal from waitlists due to health deterioration while 12 programs reported collecting data on pediatric deaths on waitlists.

### Organ preservation

Two kidney programs reported using an *ex-vivo* hypothermic perfusion pump while one program reported use of a Lifeport machine. One heart program reported having *ex-vivo* technology but not yet utilizing it, while the other reported use only for research. One lung program reported *ex-vivo* technology use. One liver program reported using OrganOx technology for organ perfusion.

### Barriers and facilitators to deceased organ donation and transplantation

When asked about other facilitators and/or barriers to donation and transplantation 3 ODOs and 6 TPs provided open ended responses.

#### ODO

Two ODOs identified culture surrounding donation in PICUs and two identified the size of the program as important barriers to pediatric organ donation and transplantation.

#### TP

Facilitators to organ donation and transplantation were identified as having ODO ccoordinators available and donation teams dedicated to PICUs. Barriers to organ donation and transplantation identified were donation culture, donor location, unavailability of ODO coordinators for timely consent.

## Discussion

Our national study demonstrated wide heterogenicity in the criteria, policies and processes for pediatric organ acceptance, offering and utilisation across Canadian ODOs and pediatric TPs. Findings from this study also demonstrated a lack of pediatric-specific programming and education across ODOs. The variation we found in practices across TPs is not only consistent with previous reports of a survey of adult kidney TPs in Canada, but extends to all pediatric solid organ TPs in Canada (19).

Variability in absolute and organ-specific offering and acceptance criteria existed across ODOs and TPs. Inconsistencies in weight and age-based exclusion criteria mean that a child of a specific weight and age may be an eligible donor in one jurisdiction but will meet absolute exclusion criteria in another. This inconsistency in practice may result in both missed donation of potentially transplantable organs and possible reluctance of care providers to approach families for organ donation when they are unsure of donation criteria. This disparity in potential donor weight criteria is more evident within the kidney TPs, with most programs excluding DCC small pediatric donors. This may be related to reports of increased incidence of graft thrombosis and loss associated with use of small pediatric kidney donors (25, 26). However, recent evidence demonstrated good renal outcome from kidneys of small pediatric donors <6 years in the first year and comparable survival with those of adult donors (26, 27). Consequently, the use of small pediatric donors may decrease the overall scarcity of kidneys for potential recipients with end stage renal disease, promote size-matching and decrease surgical complications in smaller pediatric recipients. Current practices described by Canadian ODOs and pediatric kidney TPs are not in line with current evidence. Similar differences were noticed in the donor to recipient weight discrepancy acceptance criteria within the heart TPs. While this does not necessary reflect differences in the actual practice, as TPs may decline under- or oversized donors in individual cases within the absolute exclusion limits, it may result in *a priori* non-consideration of certain donors considered suitable in other programs and national harmonization could be useful.

The finding that 6 out of 9 ODOs had not utilized potential donor organs due to lack of suitable recipients on the waitlist and 5 out of 9 reported not utilizing organs due to logistical issues is concerning given the reported mortality of children on transplant waitlists (3, 6).

Previous studies in Canada and across the world have also reported variability in specific organ allocation and utilization with high utilization rates for livers and kidneys and lower rates for hearts and lungs (13, 15–17, 28). Reasons for organ discard include older age, female gender, black race, obesity, diabetes mellitus, hypertension, hepatitis C infection and donation after cardiac death as well as high Kidney Donation Risk Index (KDRI)/Kidney Donation Profile Index (KDPI) for kidneys (29); donor's age >45 years, decreased left ventricular ejection fraction, presence of hepatitis B virus-core antibodies, hypertension, and diabetes for heart (30); increased risk primary non-function and delayed failure for liver (31–33). Furthermore, a study from the USA showed variability in organ discard rate across geographic locations, indicating that other factors apart from donor quality may contribute to the variability in organ allocation and utilizatio (29). Given the identified variability in organ offering and acceptance criteria identified in our study, it is possible that organs that were declined in one jurisdiction might have been utilized in another. This differential use of organs may create inconsistent access to donation for bereaved families and transplant for potential recipients across Canada. Standardization of organ acceptance and utilisation criteria nationally across ODOs and TPs may result in less non-utilized pediatric organs.

The development of pediatric DCC donation programs has been encouraged as a potential solution to the lack of pediatric organs for transplantation (34). However, the publishing of DCC donation guidelines and development of DCC donation programs across Canada has not yet improved pediatric donation rates (9). This is consistent with our findings that many ODOs consider organs from a DCC donor to be a barrier to organ offering and/or acceptance with a DCC donor being both an absolute and/or age-based exclusion, or a reason for non-utilization reported by many ODOs. As our results show, pediatric DCC donor status continues to be an exclusion to organ acceptance or is limited to highest urgency recipients, despite evidence of equivalent outcomes for children who receive a kidney, liver, or lung from a DNC or DCC donor (35–38). If ODOs and TPs are not accepting of DCC organ donation, developing DCC programs in PICUs and NICUs will be ineffective. Findings from our study reported low acceptance of pediatric DCC donors across TPs which is in consonance with reports across the world, ranging from 2.6% in Spain to 32.2% in the United Kigdom (10, 39–41). In contrast, reports from the USA transplant registry showed a decline in DND pediatric donors and an increase in pediatric DCC donors (11, 42). Improvement in the acceptance of pediatric DCC organs, based on evidence of equivalent outcomes for recipients, may decrease organ scarcity, time on transplant waitlist and donor-recipient size discrepancy. Creation of evidence-informed guidelines for acceptance and transplant of organs from pediatric DCC donors is essential to optimize donor potential from DCC. This is supported by emerging evidence that consistent criteria or guidelines for donation would encourage organ donation and decrease missed donation opportunities (43). The need for standardized organ acceptance, allocation and utilization guidelines nationally is further re-enforced by our finding that the majority of TPs are informed by multiple guidelines from multiple jurisdictions and that there is a lack of consistency on this use even within organs.

We also noted a lack of pediatric-specific education in many Canadian ODOs. The involvement of donation champions trained in organ donation has been shown to improve both consent and conversion rates, thus it may follow that pediatric expertise in these areas could be an asset within Canadian ODOs (8, 44, 45). Pediatric critical care is a unique environment, and communication with bereaved families of children requires specialized skills and knowledge. Educational resources for interdisciplinary teams involved with pediatric/neonatal organ donation have been created, but have not yet been widely adopted (43). National efforts for education initiatives and resource sharing between ODO's could improve this aspect of organ donation. Furthermore, the majority of ODOs (7/9) reported not having a specific pediatric/neonatal committee and only 1 reported family member involvement. ODO's could benefit from committees, including family member representation, that review and address program challenges and barriers unique to pediatric/neonatal donation.

Our survey is strengthened by high participation rate with representation from all Canadian ODOs who participate in pediatric organ donation and transplantation and over 80% of all pediatric solid organ transplant programs. Another strength of our study is the rigorous survey development by members of the pediatric donation and transplant community across Canada. Our results are limited by the self-report nature of the survey and the absence of actual referral rates for donors, consent rates, numbers of utilized donors and numbers of non-utilized organs to substantiate our findings. Given the identified challenges, an audit of pediatric deaths and donation approach, referral and consent rates is essential to further inform development of solutions. Though participants were instructed to discuss the survey with their colleagues, their responses may be more representative of their individual views and practices rather than those of the program.

## Conclusion

We identified several factors that may lead to underutilization of pediatric solid organs in Canada. Most importantly, there is marked variability in pediatric and neonatal organ offering, acceptance and utilization by ODOs and pediatric TPs across Canada and reported non-acceptance of organs from DCC donors. These inconsistencies in practices and policies and the reluctance to accept DCC organs may lead to under-utilization of pediatric organs. Collaboration between ODOs and TPs to standardize pediatric organ acceptance, offering, and sharing between provinces based on contemporary evidence has the potential to optimize pediatric organ donation and transplantation in Canada.

## Data Availability

The datasets presented in this article are not readily available because they contain identifiable information. Requests to access the datasets should be directed to laurie.lee@ahs.ca.

## Supplementary material

Supplementary Table 1 CROSS checklist.

Supplementary Data Sheet 1 Transplant Program Survey.

Supplementary Data Sheet 2 ODO Survey.

Supplementary Data Sheet 3 Supplementary Figures 1, 2.

## References

[1] Wolfe RA Ashby VB Milford EL Ojo AO Ettenger RE Agodoa LYC Comparison of mortality in all patients on dialysis, patients on dialysis awaiting transplantation, and recipients of a first cadaveric transplant. N Engl J Med. (1999) 341(23):1725–30. 10.1056/NEJM199912023412303 10580071

[2] Rabbat CG Thorpe KE Russell JD Churchill DN. Comparison of mortality risk for dialysis patients and cadaveric first renal transplant recipients in Ontario, Canada. J Am Soc Nephrol. (2000) 11(5):917–22. 10.1681/ASN.V115917 10770970

[3] Global Observatory on Donation and Transplantation. International report on organ and transplantation activities: 2021. Transplant Observatory (2022). Available online at: https://www.transplant-observatory.org/2021-global-report-5/ (Accessed January 15, 2024).

[4] Hsu E Perito ER Mazariegos G. Save the children: the ethical argument for preferential priority to minors in deceased donor liver allocation. Clin Liver Dis (Hoboken). (2021) 17(4):312–6. 10.1002/cld.1039 33968395 PMC8087936

[5] United States’ Organ Procurement and Transplantation Network (OPNT). Available online at: https://optn.transplant.hrsa.gov/data/view-data-reports/national-data/# (Accessed January 15, 2024).

[6] Husain SA King KL Pastan S Patzer RE Cohen DJ Radhakrishnan J Association between declined offers of deceased donor kidney allograft and outcomes in kidney transplant candidates. JAMA Netw Open. (2019) 2(8):e1910312. 10.1001/jamanetworkopen.2019.10312 31469394 PMC6724162

[7] Weiss MJ Pérez Blanco A Gelbart B. Special issues in pediatric deceased organ donation. Intensive Care Med. (2019) 45(3):361–3. 10.1007/s00134-019-05523-2 30725135

[8] Hawkins KC Scales A Murphy P Madden S Brierley J. Current status of paediatric and neonatal organ donation in the UK. Arch Dis Child. (2018) 103(3):210–5. 10.1136/archdischild-2017-313466 29242244

[9] Weiss MJ Hornby L Rochwerg B van Manen M Dhanani S Sivarajan VB Canadian Guidelines for controlled pediatric donation after circulatory determination of death-summary report. Pediatr Crit Care Med. (2017) 18(11):1035–46. 10.1097/PCC.0000000000001320 28925929 PMC5671796

[10] Weiss MJ Dominguez-Gil B Lahaie N Nakagawa TA Scales A Hornby L Development of a multinational registry of pediatric deceased organ donation activity. Pediatr Transplant. (2019) 23(3):e13345. 10.1111/petr.13345 30724003

[11] Workman JK Myrick CW Meyers RL Bratton SL Nakagawa TA. Pediatric organ donation and transplantation. Pediatrics. (2013) 131(6):e1723–30. 10.1542/peds.2012-3992 23690525

[12] Tsai E Shemie SD Cox PN Furst S McCarthy L Hebert D. Organ donation in children: role of the pediatric intensive care unit. Pediatr Crit Care Med. (2000) 1(2):156–60. 10.1097/00130478-200010000-00012 12813268

[13] Registry A. 2023 Annual Report. Adelaide: Australia and New Zealand Dialysis and Transplant Registry (2023).

[14] Schold JD Arrigain S Flechner SM Augustine JJ Sedor JR Wee A Dramatic secular changes in prognosis for kidney transplant candidates in the United States. Am J Transplant. (2019) 19(2):414–24. 10.1111/ajt.15021 30019832

[15] Zaroff JG Rosengard BR Armstrong WF Babcock WD D’Alessandro A Dec GW Maximizing use of organs recovered from the cadaver donor: cardiac recommendations. J Heart Lung Transplant. (2002) 21(11):1153–60. 10.1016/S1053-2498(02)00526-0

[16] United Network for Organ Sharing. Deceased organ specific donors by gender. Available online at: http://www.optn.org/latestData/rptData.asp

[17] Pierre AF Sekine Y Hutcheon MA Waddell TK Keshavjee SH. Marginal donor lungs: a reassessment. J Thorac Cardiovasc Surg. (2002) 123(3):421–7; discussion, 7–8. 10.1067/mtc.2002.120345 11882811

[18] Brennan C Husain SA King KL Tsapepas D Ratner LE Jin Z A donor utilization Index to assess the utilization and discard of deceased donor kidneys perceived as high risk. Clin J Am Soc Nephrol. (2019) 14(11):1634–41. 10.2215/CJN.02770319 31624140 PMC6832051

[19] Vinson AJ Cardinal H Parsons C Tennankore KK Mainra R Maru K Disparities in deceased donor kidney offer acceptance: a survey of Canadian transplant nephrologists, general surgeons and urologists. Can J Kidney Health Dis. (2023) 10:20543581231156855. 10.1177/20543581231156855 36861114 PMC9969426

[20] Sharma A Minh Duc NT Luu Lam Thang T Nam NH Ng SJ Abbas KS A consensus-based checklist for reporting of survey studies (CROSS). J Gen Intern Med. (2021) 36(10):3179–87. 10.1007/s11606-021-06737-1 33886027 PMC8481359

[21] Lee LA Martin DA Mahoney M James L Avitzur Y Carroll A Organ donation in Canadian PICUs: a cross-sectional survey, 2021–2022. Pediatr Crit Care Med. (2024) 25(5):416–24. 10.1097/PCC.0000000000003404 37966310 PMC11060061

[22] Burns KE Duffett M Kho ME Meade MO Adhikari NK Sinuff T A guide for the design and conduct of self-administered surveys of clinicians. CMAJ. (2008) 179(3):245–52. 10.1503/cmaj.080372 18663204 PMC2474876

[23] Vaismoradi M Turunen H Bondas T. Content analysis and thematic analysis: implications for conducting a qualitative descriptive study. Nurs Health Sci. (2013) 15(3):398–405. 10.1111/nhs.12048 23480423

[24] Elo S Kyngäs H. The qualitative content analysis process. J Adv Nurs. (2008) 62(1):107–15. 10.1111/j.1365-2648.2007.04569.x 18352969

[25] Kim JK Chua ME Teoh CW Lee MJ Kesavan A Hebert D Assessment of prophylactic heparin infusion as a safe preventative measure for thrombotic complications in pediatric kidney transplant recipients weighing <20 kg. Pediatr Transplant. (2019) 23(6):e13512. 10.1111/petr.13512 31169341

[26] Damji S Callaghan CJ Loukopoulos I Kessaris N Stojanovic J Marks SD Utilisation of small paediatric donor kidneys for transplantation. Pediatr Nephrol. (2019) 34(10):1717–26. 10.1007/s00467-018-4073-5 30238149 PMC6775037

[27] Lysakowski S Druck Garcia C Weisheimer Rohde R Pascual Vitola S Silva Pires F Carla de Souza V Pediatric kidney transplantation: outcomes with under and over 6-year-old donors. J Pediatr (Rio J). (2024) 100(1):67–73. 10.1016/j.jped.2023.07.005 37591483 PMC10751696

[28] Badovinac K. Canadian Review of organ utilization (1992–2001). Edmonton: Canadian Council for Doantion and Transplantation. (2004).

[29] Mohan S Chiles MC Patzer RE Pastan SO Husain SA Carpenter DJ Factors leading to the discard of deceased donor kidneys in the United States. Kidney Int. (2018) 94(1):187–98. 10.1016/j.kint.2018.02.016 29735310 PMC6015528

[30] Reul RM Jr. Saleem AA Keller CN Malik TH Rosengart TK Goss JA Allograft discard risk index for heart transplantation. Clin Transplant. (2021) 35(11):e14442. 10.1111/ctr.14442 34319617

[31] Ploeg RJ D'Alessandro AM Knechtle SJ Stegall MD Pirsch JD Hoffmann RM Risk factors for primary dysfunction after liver transplantation–a multivariate analysis. Transplantation. (1993) 55(4):807–13. 10.1097/00007890-199304000-00024 8475556

[32] Hsu EK Shaffer ML Gao L Sonnenday C Volk ML Bucuvalas J Analysis of liver offers to pediatric candidates on the transplant wait list. Gastroenterology. (2017) 153(4):988–95. 10.1053/j.gastro.2017.06.053 28711630 PMC6288076

[33] Ghinolfi D Lai Q Pezzati D De Simone P Rreka E Filipponi F. Use of elderly donors in liver transplantation: a paired-match analysis at a single center. Ann Surg. (2018) 268(2):325–31. 10.1097/SLA.0000000000002305 28549011

[34] Weiss MJ. The path to paediatric donation after circulatory determination of death guidelines. Paediatr Child Health. (2018) 23(1):27–30. 10.1093/pch/pxx168 29479276 PMC5815084

[35] Marlais M Pankhurst L Hudson A Sharif K Marks SD. UK national registry study of kidney donation after circulatory death for pediatric recipients. Transplantation. (2017) 101(6):1177–81. 10.1097/TP.0000000000001264 27362304

[36] Hong JC Venick R Yersiz H Kositamongkol P Kaldas FM Petrowsky H Liver transplantation in children using organ donation after circulatory death: a case-control outcomes analysis of a 20-year experience in a single center. JAMA Surg. (2014) 149(1):77–82. 10.1001/jamasurg.2013.3195 24257966

[37] MacConmara M El Mokdad A Gattineni J Hwang CS. Donation after cardiac death kidneys are suitable for pediatric recipients. Pediatr Transplant. (2019) 23(7):e13540. 10.1111/petr.13540 31278813

[38] Hwang CS Levea SL Parekh JR Liang Y Desai DM MacConmara M. Should more donation after cardiac death livers be used in pediatric transplantation? Pediatr Transplant. (2019) 23(1):e13323. 10.1111/petr.13323 30447034

[39] Koogler T Costarino AT Jr. The potential benefits of the pediatric nonheartbeating organ donor. Pediatrics. (1998) 101(6):1049–52. 10.1542/peds.101.6.1049 9606234

[40] Weiss MJ Hornby L Witteman W Shemie SD. Pediatric donation after circulatory determination of death: a scoping review. Pediatr Crit Care Med. (2016) 17(3):e87–108. 10.1097/PCC.0000000000000602 26727103

[41] Corkery-Lavender T Millar J Cavazzoni E Gelbart B. Patterns of organ donation in children in Australia and New Zealand. Crit Care Resusc. (2017) 19(4):296–302. 29202255

[42] Ghavam A Thompson NE Lee J. Comparison of pediatric brain-dead donors to donation after circulatory death donors in the United States. Pediatr Transplant. (2021) 25(3):e13926. 10.1111/petr.13926 33326666

[43] Bursac I Mtaweh H Lee D Mema B. The gift of life: interprofessional organ donation curriculum in pediatric critical care. ATS Sch. (2022) 3(1):144–55. 10.34197/ats-scholar.2021-0089IN 35634001 PMC9131884

[44] Lenzi JA Sarlo R Assis A Ponte M Paura P Araújo C Family informed consent to organ donation–who performs better: organ procurement organizations, in-hospital coordinators, or intensive care unit professionals? Transplant Proc. (2014) 46(6):1672–3. 10.1016/j.transproceed.2014.05.036 25131009

[45] Sarlo R Pereira G Surica M Almeida D Araújo C Figueiredo O Impact of introducing full-time in-house coordinators on referral and organ donation rates in Rio De Janeiro public hospitals: a health care innovation practice. Transplant Proc. (2016) 48(7):2396–8. 10.1016/j.transproceed.2015.11.044 27742307

