# Supplementary material for: Variability in pediatric and neonatal organ offering, acceptance and utilization: a survey of Canadian pediatric transplant programs and organ donation organizations
Source: Front Transplant. 2024 Sep 27;3:1458563. doi: 10.3389/frtra.2024.1458563 (PMC11466726; doi:10.3389/frtra.2024.1458563)
Supplement: Supplementary Data Sheet 1 — Transplant Program Survey. [file Datasheet1.pdf]

Supplemental File 2: Transplant Program Survey

Introduction

Pediatric Organ Donation and Transplantation Survey

Transplant Centres

The purpose of this project is to understand the current state of pediatric and neonatal organ donation and transplant of pediatric organs in Canada. We are surveying pediatric intensive care units, neonatal intensive care units, organ donation organizations and pediatric transplant programs across Canada.

Please answer the following questions based on the current state in your transplant program. Only one survey is being completed for each organ group at each center therefore, we would like the answers to be representative of your program as a whole. Please feel free to discuss answers with your colleagues to determine the answer that best aligns with practices at your program. You can save your progress at any time using the save button at the bottom of each page. You will be prompted to enter your email address to receive a link to return to the survey at any time.

Ethics ID: REB 21-0021
Study Title: Survey of Current Practices in Pediatric and Neonatal Donation and Transplantation in Canada
PI: Laurie Lee, NP
Version 1.0 07-Jan-2021

For the purposes of this survey, a transplant program has been defined as "programs involved in the acceptance and transplantation of solid organs in pediatric (0 to 18 years of age) recipients." When we ask about "program" we are referring to your organ group at your center.

Questions

[MISSING IMAGE: ,]

IMPLIED CONSENT TO PARTICIPATE IN RESEARCH

Title: Survey of Current Practices in Pediatric and Neonatal Donation and Transplantation in Canada

Funding: Canadian Blood Services

Principal Investigator:

Laurie A. Lee NP, MN Alberta Children's Hospital,

Laurie.lee@albertahealthservices.ca

403-955-2560

Co-Investigator (s):

Meagan Mahoney, MD, FRCPC, Alberta Children's Hospital, Calgary, Alberta

Meagan.Mahoney@albertahealthservices.ca

Lorraine A. Hamiwka, MD FRCPC, Alberta Children's Hospital

lorraine.hamiwka@ahs.ca

Lee James, RN, MN, Canadian Blood Services

Lee.james@blood.ca

Yaron Avitzur, MD, Division of Gastroenterology, Hepatology and Nutrition, SickKids Hospital, Toronto, ON

Yaron.Avitzur@sickkids.ca

Allison Carroll, MD, Stollery Children's Hospital

Allison.Carroll@albertahealthservices.ca

Clare Payne, Trillium Gift of Life Network

CPayne@GiftofLife.on.ca

Bailey Piggott, Canadian Blood Services

Bailey.piggott@blood.ca

Christopher Tomlinson, MBChB, PhD, Associate Professor Dept of Paediatrics University of Toronto, Neonatologist, Hospital for Sick Children, Toronto

christopher.tomlinson@sickkids.ca

Simon Urschel, MD, Associate Professor of Pediatrics and Immunology Director Pediatric Cardiac Transplantation Pediatric Cardiologist University of Alberta / Stollery Children's Hospital

urschel@ualberta.ca

Introduction

Laurie Lee, NP, MN and associates from the University of Calgary, Canadian Blood Services, the Canadian Donation and Transplantation Research Program, Trillium Gift of life Network, and Canadian Society of Transplantation are conducting a research study.

This consent form is only part of the process of informed consent. It should give you the basic idea of what the research is about and what your participation will involve. If you would like more detail about something mentioned here, or information not included here, please ask. Take the time to read this carefully and to understand any accompanying information.

You are invited to be in this study because the area you work in is vital to pediatric and/or neonatal organ donation and transplantation. Your participation in this research study is voluntary.

Why is this study being done?

Currently there is a lack of knowledge about the current state of pediatric or neonatal donation or transplant programs in Canada. Each PICU/NICU, ODO and transplant program operates in a silo despite the interrelated nature of their process and procedures, and the potential national reach of their requirements. The purpose of this study is to utilize a multi-survey approach to perform an environmental scan of all programs involved in pediatric or neonatal organ donation and transplant.

What will happen if I take part in this research study?

The survey should take approximately 20 minutes to answer and is a single survey. You do not have to answer any questions that you do not want to answer. You may withdraw at any point in the survey during its completion without impact on your personal, employment or academic status. Once the survey has been completed and submitted, you will not be able to withdraw your data. Once you have completed the survey, please press submit on your computer and it will finish. Upon completion of the survey, we will ask for your contact information to allow us to follow up on any questions we have. You do not have to provide this information. If you provide this information your contact information will be kept confidential and not included in any analysis, study results or publications.

Are there any potential risks or discomforts that I can expect from this study?

There are no known risks of participation in these surveys and no direct benefit to you. However, you will be able to openly share your opinion, your experience and your protocols in relation to pediatric and neonatal donation and transplant in Canada. There may be significant benefit to the community at large since we hope to influence further policies regarding pediatric and neonatal donation and transplantation.

WILL INFORMATION ABOUT ME AND MY PARTICIPATION BE KEPT CONFIDENTIAL?

The information that you share will remain strictly confidential and will be used solely for the purposes of this research and in accordance with all applicable privacy legislation in Alberta. The only people who will have access to the research data are the investigators. Your answers to open-ended questions may be used verbatim in presentations and publications but neither you nor your organization or hospital will be identified. Data regarding sites will be made available through internal reporting as well as peer reviewed publication.

HOW LONG WILL INFORMATION FROM THE STUDY BE KEPT?

The survey responses will be stored on the Interceptum Platform, which is licensed to and maintained by Canadian Blood Services. The servers are physically located in Montreal, Quebec. The researchers intend to keep the research data and records for approximately **5** years following publication of the results of this study.

WHOM MAY I CONTACT IF I HAVE QUESTIONS ABOUT THIS STUDY?

The Research Team:

You may contact Laurie Lee, NP at (403) 955-2560 with any questions or concerns about the research or your participation in this study.

Conjoint Health Research Ethics Board (CHREB):

If you have any questions concerning your rights as a possible participant in this research, please contact the Chair, Conjoint Health Research Ethics Board, University of Calgary at 403-220-7990.

AGREEMENT TO PARTICIPATE

Your decision to complete this survey will be interpreted as an indication of your agreement to participate. In no way does this waive your legal rights nor release the investigators or involved institutions from their legal and professional responsibilities.

Q1 Pediatric transplant program

1. For which of the following organs would your program be considered a “pediatric transplant program” based on the definition above. Please select all that apply

- ☐ Heart
- ☐ Lung
- ☐ Kidney
- ☐ Liver
- ☐ Intestine
- ☐ Pancreas

Q2 how many pediatric transplants

Show row "**Heart**" when: **Q1 Pediatric transplant program:** Heart

Show row "**Lung**" when: **Q1 Pediatric transplant program:** Lung

Show row "**Kidney**" when: **Q1 Pediatric transplant program:** Kidney

Show row "**Liver**" when: **Q1 Pediatric transplant program:** Liver

Show row "**Intestine**" when: **Q1 Pediatric transplant program:** Intestine

Show row "**Pancreas**" when: **Q1 Pediatric transplant program:** Pancreas

2. For the transplant organ you are involved with, approximately how many pediatric transplants per year would your program be responsible for?

	Pediatric Transplants				
	1-5	6-10	11-15	15-20	>20
Heart	◯	◯	◯	◯	◯
Lung	◯	◯	◯	◯	◯
Kidney	◯	◯	◯	◯	◯
Liver	◯	◯	◯	◯	◯
Intestine	◯	◯	◯	◯	◯
Pancreas	◯	◯	◯	◯	◯

Q3 catchment area

Show row "**Heart**" when: **Q1 Pediatric transplant program:** Heart

Show row "**Lung**" when: **Q1 Pediatric transplant program:** Lung

Show row "**Kidney**" when: **Q1 Pediatric transplant program:** Kidney

Show row "**Liver**" when: **Q1 Pediatric transplant program:** Liver

Show row "**Intestine**" when: **Q1 Pediatric transplant program:** Intestine

Show row "**Pancreas**" when: **Q1 Pediatric transplant program:** Pancreas

3. For the transplant program you are involved, what is the defined geographical referral catchment area? (i.e. the geographical area that patients come to your unit from)

	Please describe
Heart	
Lung	
Kidney	
Liver	
Intestine	
Pancreas	

Q4 recipient age

Show row "**Heart**" when: **Q1 Pediatric transplant program:** Heart

Show row "**Lung**" when: **Q1 Pediatric transplant program:** Lung

Show row "**Kidney**" when: **Q1 Pediatric transplant program:** Kidney

Show row "**Liver**" when: **Q1 Pediatric transplant program:** Liver

Show row "**Intestine**" when: **Q1 Pediatric transplant program:** Intestine

Show row "**Pancreas**" when: **Q1 Pediatric transplant program:** Pancreas

4. What is the age range of recipients in your transplant program?

	Minimum age	Maximum age
Heart		
Lung		
Kidney		
Liver		
Intestine		
Pancreas		

Comments

Q5 review offer

The following questions are related to organ acceptance for your transplant program.

5. At your transplant organization, who receives and reviews the organ offer?

◯ Transplant physician

◯ Transplant surgeon

◯ Both transplant physician and surgeon

◯ Other; please specify

Other - Specify

Q6 min data set

6. Do you have a minimum set of donor information that is required for each organ offer?

◯ Yes

◯ No

Q7 insufficient info

7. Is insufficient information about the donor a barrier to timely acceptance?

◯ Yes

◯ No

Q7 if yes

Complete only when:
Q7 insufficient info: Yes

If yes, which of the following are commonly missing or delayed? Please select all that apply:

Q7a Heart

Complete only when:
All criteria must be met
Q1 Pediatric transplant program: Heart
Q7 insufficient info: Yes

Heart

- [ ] Imaging
- [ ] Echo
- [ ] Angiography
- [ ] Patient History
- [ ] Laboratory parameters (including HLA and Infectious Disease information)
- [ ] Current inotropic support information
- [ ] Infectious disease testing
- [ ] Access to viewing studies (i.e. CT, ultrasound, echo, etc.)
- [ ] Other (please specify in comments)

Other - Specify

Q7b Lung

Complete only when:
All criteria must be met
Q1 Pediatric transplant program: Lung
Q7 insufficient info: Yes

Lung

- [ ] Imaging
- [ ] Bronchoscopy information
- [ ] Patient History
- [ ] Laboratory parameters (including HLA and Infectious Disease information)
- [ ] Current respiratory support information
- [ ] Access to viewing studies (i.e. CT, ultrasound, echo, etc.)
- [ ] Other (please specify in comments)

Other - Specify

Q7c Kidney

Complete only when:
All criteria must be met
Q1 Pediatric transplant program: Kidney
Q7 insufficient info: Yes

Kidney

- ☐ Imaging
- ☐ Patient History
- ☐ Laboratory parameters (including HLA and Infectious Disease information)
- ☐ Access to viewing studies (i.e. CT, ultrasound, echo, etc.)
- ☐ Other (please specify in comments)

Other - Specify

Q7d Liver

Complete only when:
All criteria must be met
Q1 Pediatric transplant program: Liver
Q7 insufficient info: Yes

Liver

- [ ] Imaging
- [ ] Patient History
- [ ] Laboratory parameters (including HLA and Infectious Disease information)
- [ ] Access to viewing studies (i.e. CT, ultrasound, echo, etc.)
- [ ] Current inotropic support information
- [ ] Other (please specify in comments)

Other - Specify

Q7e Intestine

Complete only when:
All criteria must be met
Q1 Pediatric transplant program: Intestine
Q7 insufficient info: Yes

Intestine

- [ ] Imaging
- [ ] Patient History
- [ ] Laboratory parameters (including HLA and Infectious Disease information)
- [ ] Access to viewing studies (i.e. CT, ultrasound, echo, etc.)
- [ ] Current inotropic support information
- [ ] Other (please specify in comments)

Other - Specify

Q7f Pancreas

Complete only when:
All criteria must be met
Q1 Pediatric transplant program: Pancreas
Q7 insufficient info: Yes

Pancreas

- [ ] Imaging
- [ ] Patient History
- [ ] Laboratory parameters (including HLA and Infectious Disease information)
- [ ] Access to viewing studies (i.e. CT, ultrasound, echo, etc.)
- [ ] Current inotropic support information
- [ ] Other (please specify in comments)

Other - Specify

Q8 exclusion criteria

8. Please list exclusion criteria your transplant program uses for regular urgency recipients and high urgency recipients.

Please use your site's definitions for regular urgency patients and high urgency patients.

For each of the fields below:

Describe your program's defined criteria.

If your program does not have defined criteria, please enter "N/A".

If criteria is the same for both regular and high urgency recipients, please enter your defined criteria under regular urgency and select same.

Q8a exclusion criteria Heart

Complete only when:
Q1 Pediatric transplant program: Heart

Heart

	Exclusion critera for **regular urgency recipient**	Same exclusion criteria for **high urgency recipient**	Different exclusion criteria for **high urgency recipient**
Size/ weight discrepancy of donor to recipient		undefined ☐	
Age of donor		undefined ☐	
DCD donor		undefined ☐	
Maximum time to death (warm ischemic time)		undefined ☐	
Prolonged cardiac arrest before donor declaration. Please provide threshold		undefined ☐	
HLA matching		undefined ☐	

Do you accept presence of high level Class I HLA antibodies against antigens on the donor organ?		undefined ☐	
Do you accept presence of high level Class II HLA antibodies against antigens on the donor organ?		undefined ☐	
Ejection fraction, please list minimum threshold		undefined ☐	
Recipient considerations		undefined ☐	
Donor distance (expected cold ischemic time)		undefined ☐	
Do you accept high risk donors (eg. drug use, unknown sexual history, etc)		undefined ☐	
Laboratory parameters (e.g. Troponin, CK)		undefined ☐	

Q8a heart ABO

Complete only when:
Q1 Pediatric transplant program: Heart

	Exclusion critera for **regular urgency recipient**	Same exclusion criteria for **high urgency recipient**	Different exclusion criteria for **high urgency recipient**
ABO compatibility	ABO incompatible regardless of age ☐<br>ABO incompatible (age dependent) ☐	undefined ☐	ABO incompatible regardless of age ☐<br>ABO incompatible (age dependent) ☐

If so, up to which age would you accept an ABO incompatible donor:

Q8a heart infection

Complete only when:
Q1 Pediatric transplant program: Heart

	Exclusion critera for **regular urgency recipient**	Same exclusion criteria for **high urgency recipient**	Different exclusion criteria for **high urgency recipient**
Infections	HIV ☐<br>HBV ☐<br>HCV ☐<br>West Nile ☐<br>unclear viral infection ☐<br>encephalitis ☐<br>septicemia ☐<br>other (please list below) ☐	undefined ☐	HIV ☐<br>HBV ☐<br>HCV ☐<br>West Nile ☐<br>unclear viral infection ☐<br>encephalitis ☐<br>septicemia ☐<br>other (please list below) ☐

Comments:

Q8b exclusion criteria lung

Complete only when:
Q1 Pediatric transplant program: Lung

Lung

	Exclusion critera for **regular urgency recipient**	Same exclusion criteria for **high urgency recipient**	Different exclusion criteria for **high urgency recipient**
Size/ weight discrepancy of donor to recipient		undefined ☐	
Age of donor		undefined ☐	
DCD donor		undefined ☐	
Maximum time to death (warm ischemic time)		undefined ☐	
HLA matching		undefined ☐	
Do you accept presence of high level Class I HLA antibodies against antigens on the donor organ?		undefined ☐	

Do you accept presence of high level Class II HLA antibodies against antigens on the donor organ?		undefined ☐	
Recipient considerations		undefined ☐	
Donor distance (expected cold ischemic time)		undefined ☐	
Do you accept high risk donors (eg. drug use, unknown sexual history, etc)		undefined ☐	
Laboratory parameters (e.g. Troponin, CK)		undefined ☐	
Organ function/quality		undefined ☐	

Q8b lung ABO

Complete only when:
Q1 Pediatric transplant program: Lung

	Exclusion critera for **regular urgency recipient**	Same exclusion criteria for **high urgency recipient**	Different exclusion criteria for **high urgency recipient**
ABO compatibility	ABO incompatible regardless of age ☐<br>ABO incompatible (age dependent) ☐	undefined ☐	ABO incompatible regardless of age ☐<br>ABO incompatible (age dependent) ☐

If age dependent, please describe age based exclusion criteria for ABO incompatible organs:

Q8b lung infection

Complete only when:
Q1 Pediatric transplant program: Lung

	Exclusion critera for **regular urgency recipient**		Same exclusion criteria for **high urgency recipient**	Different exclusion criteria for **high urgency recipient**	
Infections	HIV	☐	undefined ☐	HIV	☐
	HBV	☐		HBV	☐
	HCV	☐		HCV	☐
	West Nile	☐		West Nile	☐
	unclear viral infection	☐		unclear viral infection	☐
	encephalitis	☐		encephalitis	☐
	septicemia	☐		septicemia	☐
	other (please list below)	☐		other (please list below)	☐

Comments:

Q8c exclusion criteria kidney

Complete only when:
Q1 Pediatric transplant program: Kidney

Kidney

	Exclusion critera for **regular urgency recipient**	Same exclusion criteria for **high urgency recipient**	Different exclusion criteria for **high urgency recipient**
Size/ weight discrepancy of donor to recipient		undefined ☐	
Age of donor		undefined ☐	
DCD donor		undefined ☐	
Maximum time to death (warm ischemic time)		undefined ☐	
HLA matching		undefined ☐	
Do you accept presence of high level Class I HLA antibodies against antigens on the donor organ?		undefined ☐	

Do you accept presence of high level Class II HLA antibodies against antigens on the donor organ?		undefined ☐	
Recipient considerations		undefined ☐	
Donor distance (expected cold ischemic time)		undefined ☐	
Do you accept high risk donors (eg. drug use, unknown sexual history, etc)		undefined ☐	
Laboratory parameters (e.g. Troponin, CK)		undefined ☐	
Organ function (eg. current or maximum creatinine, urine studies)		undefined ☐	

Q8c kidney ABO

Complete only when:
Q1 Pediatric transplant program: Kidney

	Exclusion critera for **regular urgency recipient**	Same exclusion criteria for **high urgency recipient**	Different exclusion criteria for **high urgency recipient**
ABO compatibility	ABO incompatible regardless of age ☐<br>ABO incompatible (age dependent) ☐	undefined ☐	ABO incompatible regardless of age ☐<br>ABO incompatible (age dependent) ☐

If so, up to which age would you accept an ABO incompatible donor:

Q8c kidney infection

Complete only when:
Q1 Pediatric transplant program: Kidney

	Exclusion critera for **regular urgency recipient**	Same exclusion criteria for **high urgency recipient**	Different exclusion criteria for **high urgency recipient**
Infections	HIV ☐<br>HBV ☐<br>HCV ☐<br>West Nile ☐<br>unclear viral infection ☐<br>encephalitis ☐<br>septicemia ☐<br>other (please list below) ☐	undefined ☐	HIV ☐<br>HBV ☐<br>HCV ☐<br>West Nile ☐<br>unclear viral infection ☐<br>encephalitis ☐<br>septicemia ☐<br>other (please list below) ☐

Comments:

Q8d exclusion criteria liver

Complete only when:
Q1 Pediatric transplant program: Liver

Liver

	Exclusion critera for **regular urgency recipient**	Same exclusion criteria for **high urgency recipient**	Different exclusion criteria for **high urgency recipient**
Size/ weight discrepancy of donor to recipient		undefined ☐	
Age of donor		undefined ☐	
DCD donor		undefined ☐	
Maximum time to death (warm ischemic time)		undefined ☐	
HLA matching		undefined ☐	
Do you accept presence of high level Class I HLA antibodies against antigens on the donor organ?		undefined ☐	

Do you accept presence of high level Class II HLA antibodies against antigens on the donor organ?		undefined ☐	
Recipient considerations		undefined ☐	
Donor distance (expected cold ischemic time)		undefined ☐	
Do you accept high risk donors (eg. drug use, unknown sexual history, etc)		undefined ☐	
Laboratory parameters (e.g. Troponin, CK)		undefined ☐	
Organ function		undefined ☐	

Q8d liver ABO

Complete only when:
Q1 Pediatric transplant program: Liver

	Exclusion critera for **regular urgency recipient**	Same exclusion criteria for **high urgency recipient**	Different exclusion criteria for **high urgency recipient**
ABO compatibility	ABO incompatible regardless of age ☐<br>ABO incompatible (age dependent) ☐	undefined ☐	ABO incompatible regardless of age ☐<br>ABO incompatible (age dependent) ☐

If so, up to which age would you accept an ABO incompatible donor:

Q8d liver infection

Complete only when:
Q1 Pediatric transplant program: Liver

	Exclusion critera for **regular urgency recipient**	Same exclusion criteria for **high urgency recipient**	Different exclusion criteria for **high urgency recipient**
Infections	HIV ☐<br>HBV ☐<br>HCV ☐<br>West Nile ☐<br>unclear viral infection ☐<br>encephalitis ☐<br>septicemia ☐<br>other (please list below) ☐	undefined ☐	HIV ☐<br>HBV ☐<br>HCV ☐<br>West Nile ☐<br>unclear viral infection ☐<br>encephalitis ☐<br>septicemia ☐<br>other (please list below) ☐

Comments:

Q8e exclusion criteria intestine

Complete only when:
Q1 Pediatric transplant program: Intestine

Intestine

	Exclusion critera for **regular urgency recipient**	Same exclusion criteria for **high urgency recipient**	Different exclusion criteria for **high urgency recipient**
Size/ weight discrepancy of donor to recipient		undefined ☐	
Age of donor		undefined ☐	
DCD donor		undefined ☐	
Maximum time to death (warm ischemic time)		undefined ☐	
HLA matching		undefined ☐	
Do you accept presence of high level Class I HLA antibodies against antigens on the donor organ?		undefined ☐	

Do you accept presence of high level Class II HLA antibodies against antigens on the donor organ?		undefined ☐	
Recipient considerations		undefined ☐	
Donor distance (expected cold ischemic time)		undefined ☐	
Do you accept high risk donors (eg. drug use, unknown sexual history, etc)		undefined ☐	
Laboratory parameters (e.g. Troponin, CK)		undefined ☐	
Organ function		undefined ☐	

Q8e intestine ABO

Complete only when:
Q1 Pediatric transplant program: Intestine

	Exclusion critera for **regular urgency recipient**	Same exclusion criteria for **high urgency recipient**	Different exclusion criteria for **high urgency recipient**
ABO compatibility	ABO incompatible regardless of age ☐<br>ABO incompatible (age dependent) ☐	undefined ☐	ABO incompatible regardless of age ☐<br>ABO incompatible (age dependent) ☐

If so, up to which age would you accept an ABO incompatible donor:

Q8e intestine infection

Complete only when:

Q1 Pediatric transplant program: Intestine

	Exclusion critera for **regular urgency recipient**	Same exclusion criteria for **high urgency recipient**	Different exclusion criteria for **high urgency recipient**
Infections	HIV ☐<br>HBV ☐<br>HCV ☐<br>West Nile ☐<br>unclear viral infection ☐<br>encephalitis ☐<br>septicemia ☐<br>other (please list below) ☐	undefined ☐	HIV ☐<br>HBV ☐<br>HCV ☐<br>West Nile ☐<br>unclear viral infection ☐<br>encephalitis ☐<br>septicemia ☐<br>other (please list below) ☐

Comments:

Q8f exclusion criteria pancreas

Complete only when:
Q1 Pediatric transplant program: Pancreas

Pancreas

	Exclusion critera for **regular urgency recipient**	Same exclusion criteria for **high urgency recipient**	Different exclusion criteria for **high urgency recipient**
Size/ weight discrepancy of donor to recipient		undefined ☐	
Age of donor		undefined ☐	
DCD donor		undefined ☐	
Maximum time to death (warm ischemic time)		undefined ☐	
HLA matching		undefined ☐	
Do you accept presence of high level Class I HLA antibodies against antigens on the donor organ?		undefined ☐	

Do you accept presence of high level Class II HLA antibodies against antigens on the donor organ?		undefined ☐	
Recipient considerations		undefined ☐	
Donor distance (expected cold ischemic time)		undefined ☐	
Do you accept high risk donors (eg. drug use, unknown sexual history, etc)		undefined ☐	
Laboratory parameters (e.g. Troponin, CK)		undefined ☐	
Organ function		undefined ☐	

Q8f pancreas ABO

Complete only when:
Q1 Pediatric transplant program: Pancreas

	Exclusion critera for **regular urgency recipient**	Same exclusion criteria for **high urgency recipient**	Different exclusion criteria for **high urgency recipient**
ABO compatibility	ABO incompatible regardless of age ☐<br>ABO incompatible (age dependent) ☐	undefined ☐	ABO incompatible regardless of age ☐<br>ABO incompatible (age dependent) ☐

If so, up to which age would you accept an ABO incompatible donor:

Q8f pancreas infection

Complete only when:
Q1 Pediatric transplant program: Pancreas

	Exclusion critera for **regular urgency recipient**	Same exclusion criteria for **high urgency recipient**	Different exclusion criteria for **high urgency recipient**
Infections	HIV ☐<br>HBV ☐<br>HCV ☐<br>West Nile ☐<br>unclear viral infection ☐<br>encephalitis ☐<br>septicemia ☐<br>other (please list below) ☐	undefined ☐	HIV ☐<br>HBV ☐<br>HCV ☐<br>West Nile ☐<br>unclear viral infection ☐<br>encephalitis ☐<br>septicemia ☐<br>other (please list below) ☐

Comments:

Q10 accept DCD organs

Show row "**Heart**" when: **Q1 Pediatric transplant program:** Heart

Show row "**Lung**" when: **Q1 Pediatric transplant program:** Lung

Show row "**Kidney**" when: **Q1 Pediatric transplant program:** Kidney

Show row "**Liver**" when: **Q1 Pediatric transplant program:** Liver

Show row "**Intestine**" when: **Q1 Pediatric transplant program:** Intestine

Show row "**Pancreas**" when: **Q1 Pediatric transplant program:** Pancreas

10. Does your program accept DCD organs for pediatric recipients? (select all that apply)

	Yes, pediatric DCD organs	Yes, adult DCD organs	No we do not accept any DCD organs for pediatric recipients
Heart	undefined☐	undefined☐	undefined☐
Lung	undefined☐	undefined☐	undefined☐
Kidney	undefined☐	undefined☐	undefined☐
Liver	undefined☐	undefined☐	undefined☐
Intestine	undefined☐	undefined☐	undefined☐
Pancreas	undefined☐	undefined☐	undefined☐

Q10a Heart

Complete only when:
Q10 accept DCD organs:

Heart

If yes, please list the characteristics of patients for which you would accept a DCD organ:

Q10b Lung

Complete only when:
Q10 accept DCD organs:

Lung

If yes, please list the characteristics of patients for which you would accept a DCD organ:

Q10c Kidney

Complete only when:
Q10 accept DCD organs:

Kidney

If yes, please list the characteristics of patients for which you would accept a DCD organ:

Q10d Liver

Complete only when:
Q10 accept DCD organs:

Liver

If yes, please list the characteristics of patients for which you would accept a DCD organ:

Q10e Intestine

Complete only when:
Q10 accept DCD organs:

Intestine

If yes, please list the characteristics of patients for which you would accept a DCD organ:

Q10f Pancreas/Islet Cells

Complete only when:
Q10 accept DCD organs:

Pancreas/Islet Cells

If yes, please list the characteristics of patients for which you would accept a DCD organ:

Q11 ex-vivo

11. Are you using ex-vivo or other similar technology for your organ group?

◯ Yes, please describe

◯ No

Comments

Q12 allocation practice

The following questions are related to organ allocation for your transplant program.

12. Please select from below which of the following items inform allocation practices/offers of organs from pediatric donors within your program:

Q12a Heart

Complete only when:
Q1 Pediatric transplant program: Heart

Heart

☐ National allocation policy

☐ Local allocation policy

☐ Informal allocation guideline

- [ ] Expert discussion
- [ ] A-priori patient list
- [ ] Other (please specify in comments)

Other - Specify

Q12b Lung

Complete only when:
Q1 Pediatric transplant program: Lung

Lung

- [ ] National allocation policy
- [ ] Local allocation policy
- [ ] Informal allocation guideline
- [ ] Expert discussion
- [ ] A-priori patient list
- [ ] Other (please specify in comments)

Other - Specify

Q12c Kidney

Complete only when:
Q1 Pediatric transplant program: Kidney

Kidney

- [ ] National allocation policy
- [ ] Local allocation policy
- [ ] Informal allocation guideline
- [ ] Expert discussion
- [ ] A-priori patient list
- [ ] Other (please specify in comments)

Other - Specify

Q12d Liver

Complete only when:
Q1 Pediatric transplant program: Liver

Liver

- [ ] National allocation policy
- [ ] Local allocation policy

- [ ] Informal allocation guideline
- [ ] Expert discussion
- [ ] A-priori patient list
- [ ] Other (please specify in comments)

Other - Specify

Q12e Intestine

Complete only when:
Q1 Pediatric transplant program: Intestine

Intestine

- [ ] National allocation policy
- [ ] Local allocation policy
- [ ] Informal allocation guideline
- [ ] Expert discussion
- [ ] A-priori patient list
- [ ] Other (please specify in comments)

Other - Specify

Q12f Pancreas/Islet

Complete only when:
Q1 Pediatric transplant program: Pancreas

Pancreas/Islet Cells

- [ ] National allocation policy
- [ ] Local allocation policy
- [ ] Informal allocation guideline
- [ ] Expert discussion
- [ ] A-priori patient list
- [ ] Other (please specify in comments)

Other - Specify

Q13 allocation policy

13. Does your program have a policy for allocation of pediatric organs?

- ○ Yes, please share in the document sharing section below
- ○ No, please describe how you allocate pediatric organs

13b file upload

Complete only when:
Q13 allocation policy: Yes, please share in the document sharing section below

1.

2.

3.

If you have more than 3 files, please enter you email address and we will contact you:

Email

Q14 do documents accurately describe program

Complete only when:
Q13 allocation policy: Yes, please share in the document sharing section below

Show row "**Heart**" when: **Q1 Pediatric transplant program:** Heart

Show row "**Lung**" when: **Q1 Pediatric transplant program:** Lung

Show row "**Kidney**" when: **Q1 Pediatric transplant program:** Kidney

Show row "**Liver**" when: **Q1 Pediatric transplant program:** Liver

Show row "**Intestine**" when: **Q1 Pediatric transplant program:** Intestine

Show row "**Pancreas**" when: **Q1 Pediatric transplant program:** Pancreas

14. Do the allocation documents provided accurately describe your program's practices regarding prioritization of pediatric organs and recipients in comparision to adult recipients?

	Yes/No	
	Yes	No
Heart	○	○
Lung	○	○
Kidney	○	○
Liver	○	○
Intestine	○	○

Pancreas	○ ○

14b describe allocation

Complete only when:
Any criterion can be met
Q13 allocation policy: No, please describe how you allocate pediatric organs
Q14 do documents accurately describe program: [Heart][Yes/No] No
[Lung][Yes/No] No
[Kidney][Yes/No] No
[Liver][Yes/No] No
[Intestine][Yes/No] No
[Pancreas][Yes/No] No

Show row "**Heart**" when: **Q1 Pediatric transplant program:** Heart

Show row "**Lung**" when: **Q1 Pediatric transplant program:** Lung

Show row "**Kidney**" when: **Q1 Pediatric transplant program:** Kidney

Show row "**Liver**" when: **Q1 Pediatric transplant program:** Liver

Show row "**Intestine**" when: **Q1 Pediatric transplant program:** Intestine

Show row "**Pancreas**" when: **Q1 Pediatric transplant program:** Pancreas

	Please describe how you program prioritizes allocation of pediatric organs between **regular urgency** adult and pediatric recipients.	Please describe how you program prioritizes allocation of pediatric organs between **high urgency** adult and pediatric recipients.
Heart		
Lung		
Kidney		
Liver		

Intestine		
Pancreas		

Q15 database

The following questions are related to data collection for your transplant program.

15. Does your program have your own transplant database?

○ Yes

○ No

Q16 registries

16. Does your program report to any local, national or international registries?

○ Yes, please list in comments

○ No

Comments

Q17 pediatric specific data

17. Does your program collect data specifically related to pediatric transplants (i.e. offers of pediatric organs, number of pediatric organs transplanted, etc. ?

○ Yes

○ No

Q18 waitlist deaths

18. Does your program record information on death of pediatric patients on the transplant waitlist?

- ◯ Yes
- ◯ No

Q19 waitlist removal

19. Does your program record information on removal from the transplant waitlist for deterioration/too sick?

- ◯ Yes
- ◯ No

Q20 post transplant outcomes

20. How does your program collect short and long term data to follow post-transplant outcomes? Please select all_that apply:

- ☐ National database
- ☐ Provincial database
- ☐ Local/hospital database
- ☐ Paper/manual flowsheets
- ☐ International database
- ☐ Other - Please specify

- [ ] None

Other - Specify

Q21 organs declined

The following questions are related to other facilitators and barriers to pediatric donation and transplant.

21. In the last 5 years has your program declined pediatric donor organs due to any of the following logistical/surgical resource limitations?

- [ ] Lack of available retrieval staff
- [ ] Lack of OR personnel (i.e. RNs, etc.)
- [ ] Lack of OR space availability
- [ ] Lack of timely transport available for the donor organ
- [ ] Lack of timely transport available for the recipient
- [ ] Financial restrictions
- [ ] Recipient location
- [ ] Lack of patients on waitlist

☐ Other (please specify in comments)

☐ Not applicable – we have not had to decline pediatric organs due to logistical/surgical resource limitations

Other - Specify

Q22 awaiting transplant

Show statement "**Heart**" when: **Q1 Pediatric transplant program:** Heart

Show statement "**Lung**" when: **Q1 Pediatric transplant program:** Lung

Show statement "**Kidney**" when: **Q1 Pediatric transplant program:** Kidney

Show statement "**Liver**" when: **Q1 Pediatric transplant program:** Liver

Show statement "**Intestine**" when: **Q1 Pediatric transplant program:** Intestine

Show statement "**Pancreas/Islet Cells**" when: **Q1 Pediatric transplant program:** Pancreas

22. Does your program require patients to stay within a maximum distance from the transplant center while awaiting transplant?

	Yes	No
Heart	○	○
Lung	○	○
Kidney	○	○
Liver	○	○
Intestine	○	○
Pancreas/Islet Cells	○	○

22b max distance from transplant center

Complete only when:
Q22 awaiting transplant: [Heart] Yes
[Lung] Yes
[Kidney] Yes
[Liver] Yes
[Intestine] Yes
[Pancreas/Islet Cells] Yes

Show row "**Heart**" when: All criteria must be met
Q1 Pediatric transplant program: Heart
Q22 awaiting transplant: [Heart] Yes

Show row "**Lung**" when: All criteria must be met
Q1 Pediatric transplant program: Lung
Q22 awaiting transplant: [Lung] Yes

Show row "**Kidney**" when: All criteria must be met
Q1 Pediatric transplant program: Kidney
Q22 awaiting transplant: [Kidney] Yes

Show row "**Liver**" when: All criteria must be met
Q1 Pediatric transplant program: Liver
Q22 awaiting transplant: [Liver] Yes

Show row "**Intestine**" when: All criteria must be met
Q1 Pediatric transplant program: Intestine
Q22 awaiting transplant: [Intestine] Yes

Show row "**Pancreas**" when: All criteria must be met
Q1 Pediatric transplant program: Pancreas
Q22 awaiting transplant: [Pancreas/Islet Cells] Yes

	Maximum distance **regular urgency** recipients may live from the transplant center	Maximum distance **high urgency** recipients may live from the transplant center
Heart		

Lung		
Kidney		
Liver		
Intestine		
Pancreas		

Q23 pediatric guidelines

Show statement "**Heart**" when: **Q1 Pediatric transplant program:** Heart

Show statement "**Lung**" when: **Q1 Pediatric transplant program:** Lung

Show statement "**Kidney**" when: **Q1 Pediatric transplant program:** Kidney

Show statement "**Liver**" when: **Q1 Pediatric transplant program:** Liver

Show statement "**Intestine**" when: **Q1 Pediatric transplant program:** Intestine

Show statement "**Pancreas/Islet Cells**" when: **Q1 Pediatric transplant program:** Pancreas

23. Does your program have a pediatric transplant specific recipient management guideline?

	Yes	No
Heart	◯	◯
Lung	◯	◯
Kidney	◯	◯
Liver	◯	◯
Intestine	◯	◯
Pancreas/Islet Cells	◯	◯

Q24 other facilitators

24. Are there any facilitators to pediatric/neonatal organ donation within your organization that we have not asked about?

- ◯ Yes, please describe
- ◯ No

Comments

Q25 other barriers

25. Are there any barriers to pediatric/neonatal organ donation within your organization that we have not asked about?

- ◯ Yes, please describe
- ◯ No

Comments

Q26 contact info

Thank you for completing the survey.

We ask that you provide contact information below, for a member of our team to be able to contact you should we require further clarification on one of your survey answers. This information will not be shared.

If you have any questions, please contact Laurie.lee@albertahealthservices.ca

Name

Email

Phone number
