# Supplementary material for: Variability in pediatric and neonatal organ offering, acceptance and utilization: a survey of Canadian pediatric transplant programs and organ donation organizations
Source: Front Transplant. 2024 Sep 27;3:1458563. doi: 10.3389/frtra.2024.1458563 (PMC11466726; doi:10.3389/frtra.2024.1458563)
Supplement: Supplementary Data Sheet 2 — ODO Survey. [file Datasheet2.pdf]

Supplemental File 3: Organ Donation Organization Survey

Introduction

Pediatric Organ Donation and Transplantation Survey

ODO Survey

The purpose of this project is to understand the current state of pediatric and neonatal organ donation and transplant in Canada. We are surveying pediatric intensive care units, neonatal intensive care units, organ donation organizations and pediatric transplant programs across Canada.

Please answer the following questions based on current practices in your ODO. Only one survey is being completed for each ODO therefore we would like the answers to be representative of your program as a whole. Please feel free to discuss answers with your colleagues or forward the survey on to the appropriate person to ensure that questions are answered to best align with practices in your program. You can save your progress at any time using the save button at the bottom of each page. You will be prompted to enter your email address to receive a link to return to the survey at any time.

Ethics ID: REB 21-0021
Study Title: Survey of Current Practices in Pediatric and Neonatal Donation and Transplantation in Canada
PI: Laurie Lee, NP
Version 1.0 07-Jan-2021

Questions

[MISSING IMAGE: ,]

IMPLIED CONSENT TO PARTICIPATE IN RESEARCH

Title: Survey of Current Practices in Pediatric and Neonatal Donation and Transplantation in Canada

Funding: Canadian Blood Services

Principal Investigator:

Laurie A. Lee NP, MN Alberta Children's Hospital,

Laurie.lee@albertahealthservices.ca

403-955-2560

Co-Investigator (s):

Meagan Mahoney, MD, FRCPC, Alberta Children's Hospital, Calgary, Alberta

Meagan.Mahoney@albertahealthservices.ca

Lorraine A. Hamiwka, MD FRCPC, Alberta Children's Hospital

lorraine.hamiwka@ahs.ca

Lee James, RN, MN, Canadian Blood Services

Lee.james@blood.ca

Yaron Avitzur, MD, Division of Gastroenterology, Hepatology and Nutrition, SickKids Hospital, Toronto, ON

Yaron.Avitzur@sickkids.ca

Allison Carroll, MD, Stollery Children's Hospital

Allison.Carroll@albertahealthservices.ca

Clare Payne, Trillium Gift of Life Network

CPayne@GiftofLife.on.ca

Bailey Piggott, Canadian Blood Services

Bailey.piggott@blood.ca

Christopher Tomlinson, MBChB, PhD, Associate Professor Dept of Paediatrics University of Toronto, Neonatologist, Hospital for Sick Children, Toronto

christopher.tomlinson@sickkids.ca

Simon Urschel, MD, Associate Professor of Pediatrics and Immunology Director Pediatric Cardiac Transplantation Pediatric Cardiologist University of Alberta / Stollery Children's Hospital

urschel@ualberta.ca

Introduction

Laurie Lee, NP, MN and associates from the University of Calgary, Canadian Blood Services, the Canadian Donation and Transplantation Research Program, Trillium Gift of life Network, and Canadian Society of Transplantation are conducting a research study.

This consent form is only part of the process of informed consent. It should give you the basic idea of what the research is about and what your participation will involve. If you would like more detail about something mentioned here, or information not included here, please ask. Take the time to read this carefully and to understand any accompanying information.

You are invited to be in this study because the area you work in is vital to pediatric and/or neonatal organ donation and transplantation. Your participation in this research study is voluntary.

Why is this study being done?

Currently there is a lack of knowledge about the current state of pediatric or neonatal donation or transplant programs in Canada. Each PICU/NICU, ODO and transplant program operates in a silo despite the interrelated nature of their process and procedures, and the potential national reach of their requirements. The purpose of this study is to utilize a multi-survey approach to perform an environmental scan of all programs involved in pediatric or neonatal organ donation and transplant.

What will happen if I take part in this research study?

The survey should take approximately 20-30 minutes to answer and is a single survey. You do not have to answer any questions that you do not want to answer. You may withdraw at any point in the survey during its completion without impact on your personal, employment or academic status. Once the survey has been completed and submitted, you will not be able to withdraw your data. Once you have completed the survey, please press submit on your computer and it will finish. Upon completion of the survey we will ask for your contact information to allow us to follow up on any questions we have. You do not have to provide this information. If you provide this information your contact information will be kept confidential and not included in any analysis, study results or publications.

Are there any potential risks or discomforts that I can expect from this study?

There are no known risks of participation in these surveys and no direct benefit to you. However, you will be able to openly share your opinion, your experience and your protocols in relation to pediatric and neonatal donation and transplant in Canada. There may be significant benefit to the community at large since we hope to influence further policies regarding pediatric and neonatal donation and transplantation.

WILL INFORMATION ABOUT ME AND MY PARTICIPATION BE KEPT CONFIDENTIAL?

The information that you share will remain strictly confidential and will be used solely for the purposes of this research and in accordance with all applicable privacy legislation in Alberta. The only people who will have access to the research data are the investigators. Your answers to open-ended questions may be used verbatim in presentations and publications but neither you nor your organization or hospital will be identified. Data regarding sites will be made available through internal reporting as well as peer reviewed publication.

HOW LONG WILL INFORMATION FROM THE STUDY BE KEPT?

The survey responses will be stored on the Interceptum Platform, which is licensed to and maintained by Canadian Blood Services. The servers are physically located in Montreal, Quebec. The researchers intend to keep the research data and records for approximately **5** years following publication of the results of this study.

WHOM MAY I CONTACT IF I HAVE QUESTIONS ABOUT THIS STUDY?

The Research Team:

You may contact Laurie Lee, NP at (403) 955-2560 with any questions or concerns about the research or your participation in this study.

Conjoint Health Research Ethics Board (CHREB):

If you have any questions concerning your rights as a possible participant in this research, please contact the Chair, Conjoint Health Research Ethics Board, University of Calgary at 403-220-7990.

AGREEMENT TO PARTICIPATE

Your decision to complete this survey will be interpreted as an indication of your agreement to participate. In no way does this waive your legal rights nor release the investigators or involved institutions from their legal and professional responsibilities.

Q1/Q2 ODO

1. What is the name of your organ donation organization?

2. Please describe the geographical area for which your Organ Donation Organization is responsible for organ donation?

Q3 organ transplant

3. Please describe the geographical area for which your Organ Donation Organization is responsible for organ **transplant**?

- ◯ It is the same as for donation
- ◯ It is different than donation

If different, please describe:

Q4 NDD

The following questions are related to *donor acceptance practices* for pediatric (0-18 years of age) donors for your ODO.

4. For each of the organs listed below, please indicate where you offer organs from pediatric (0-18 years of age) donors. (select all that apply)

NDD Donors

	Provincially	Nationally	Internationally	All	Neither
Heart	undefined☐	undefined ☐	undefined☐	undefined ☐	undefined ☐
Lung	undefined☐	undefined ☐	undefined☐	undefined ☐	undefined ☐
Kidney	undefined☐	undefined ☐	undefined☐	undefined ☐	undefined ☐
Liver	undefined☐	undefined ☐	undefined☐	undefined ☐	undefined ☐
Intestine	undefined☐	undefined ☐	undefined☐	undefined ☐	undefined ☐
Pancreas/Islet Cells	undefined☐	undefined ☐	undefined☐	undefined ☐	undefined ☐

Other (please list in comments)	undefined ☐	undefined ☐	undefined ☐	undefined ☐	undefined ☐

Other - Specify

Q4b DCD

DCD Donors

	Provincially	Nationally	Internationally	All	Neither
Heart	undefined☐	undefined ☐	undefined☐	undefined ☐	undefined ☐
Lung	undefined☐	undefined ☐	undefined☐	undefined ☐	undefined ☐
Kidney	undefined☐	undefined ☐	undefined☐	undefined ☐	undefined ☐
Liver	undefined☐	undefined ☐	undefined☐	undefined ☐	undefined ☐
Intestine	undefined☐	undefined ☐	undefined☐	undefined ☐	undefined ☐
Pancreas/Islet Cells	undefined☐	undefined ☐	undefined☐	undefined ☐	undefined ☐
Other (please list in comments)	undefined☐	undefined ☐	undefined☐	undefined ☐	undefined ☐

Other - Specify

Q5 exclusion for any donation

5. For pediatric donors, please list any absolute specific donor exclusion criteria for **any donation**.

	Absolute specific donor exclusion criteria
Chronological age	
Weight (kg)	
Corrected gestational age for preterm infants (weeks)	
Donor distance (cold ischemic time) (hours)	
Other (please specify)	

Q6 exclusion for specific organs

6. For pediatric donors, please list any absolute specific exclusion donor criteria for **specific organs**.

	Heart	Lung	Kidney	Liver	Intestine	Pancreas/Islet Cells
Chronological age						
Weight (kg)						
Corrected gestational age for preterm infants (weeks)						
Donor distance (cold ischemic time) (hours)						
Other (please specify)						

7a offering standard organs

7a. At what point in the Organ Donation process does your program offer out/place interest calls for pediatric organs that are deemed **standard organs** by your organization?

- ◯ Only After Consent is obtained and work up is complete
- ◯ After Consent is obtained and before work up is complete
- ◯ After approach, but before consent
- ◯ Before approach to determine if there is any interest in organs
- ◯ Other, please describe

Other - Specify

____

7b offering hard to place organs

7b. At what point in the Organ Donation process does your program offer out/place interest calls for pediatric organs that are deemed **hard to place organs** by your organization?

- ◯ Only After Consent is obtained and work up is complete
- ◯ After Consent is obtained and before work up is complete
- ◯ After approach, but before consent
- ◯ Before approach to determine if there is any interest in organs
- ◯ Other, please describe

Other - Specify

____

Q8 local offers

8. What is the method of communication used by your organization to offer pediatric organs locally (within your ODO)? Select all that apply.

- [ ] Text message
- [ ] Email
- [ ] Phone call
- [ ] CTR and National organ waitlist
- [ ] iTransplant for sharing/transmitting donor chart
- [ ] Data base like system (other than iTransplant)
- [ ] Fax
- [ ] Other

Other - Specify

Q9 national offers

9. What is the method of communication used by your organization to offer pediatric organs nationally? Select all that apply.

- [ ] Text message
- [ ] Email
- [ ] Phone call

- [ ] CTR and National organ waitlist
- [ ] iTransplant for sharing/transmitting donor chart
- [ ] Data base like system (other than iTransplant)
- [ ] Fax
- [ ] Other

Other - Specify

Q10 not utilized organs

10. In the past 3 years, have you had organs that are not utilized due to lack of recipients on the waitlist?

○ Yes, please list which organs and any common characteristics of the organs that are declined due to lack of recipients on the wait list

○ No

Comments

Q11 not utilized organs - not waitlist

11. In the past 3 years, have you had organs that are not utilized for other reasons than lack of recipients on the waitlist?

○ Yes, please describe below

○ No

Comments

Q12 retrieval

The following questions are related to organ recovery.

12. Please select the option that best describes your transplant programs in terms of what local personnel/retrieval capabilities are available for pediatric donors of each of the following organs.

	We have local organ recovery personnel/retrieval teams that are able to recover organs from ALL pediatric donors regardless of age	Our local organ recovery personnel/retrieval teams are ONLY able to recover organs from larger pediatric donors (ex. >10kg or >1 year of age) Please list age, weight, or other cut offs below	We DO NOT have local organ recovery personnel/retrieval that are able to recover organs from ANY pediatric patients
Heart	○	○	○
Lungs	○	○	○
Kidney	○	○	○
Liver	○	○	○

Intestine	○	○	○
Pancreas/ Islet Cells	○	○	○

12a heart

Complete only when:
Q12 retrieval: [Heart] Our local organ recovery personnel/retrieval teams are ONLY able to recover organs from larger pediatric donors (ex. >10kg or >1 year of age) Please list age, weight, or other cut offs below

Heart - age, weight, or other cut offs:

12b lungs

Complete only when:
Q12 retrieval: [Lungs] Our local organ recovery personnel/retrieval teams are ONLY able to recover organs from larger pediatric donors (ex. >10kg or >1 year of age) Please list age, weight, or other cut offs below

Lungs - age, weight, or other cut offs:

12c Kidney

Complete only when:
Q12 retrieval: [Kidney] Our local organ recovery personnel/retrieval teams are ONLY able to recover organs from larger pediatric donors (ex. >10kg or >1 year of age) Please list age, weight, or other cut offs below

Kidney - age, weight, or other cut offs:

12d Liver

Complete only when:
Q12 retrieval: [Liver] Our local organ recovery personnel/retrieval teams are ONLY able to recover organs from larger pediatric donors (ex. >10kg or >1 year of age) Please list age, weight, or other cut offs below

Liver - age, weight, or other cut offs:

12e Intestine

Complete only when:
Q12 retrieval: [Intestine] Our local organ recovery personnel/retrieval teams are ONLY able to recover organs from larger pediatric donors (ex. >10kg or >1 year of age) Please list age, weight, or other cut offs below

Intestine - age, weight, or other cut offs:

12f pancreas/islet cells

Complete only when:
Q12 retrieval: [Pancreas/ Islet Cells] Our local organ recovery personnel/retrieval teams are ONLY able to recover organs from larger pediatric donors (ex. >10kg or >1 year of age) Please list age, weight, or other cut offs below

Pancreas/Islet cells - age, weight, or other cut offs:

Q13 who performs retrieval

13. Please select who of the following most commonly performs organ retrieval for your program?

- ◯ Staff Surgeon
- ◯ Surgical fellow
- ◯ Surgical Resident
- ◯ Technician

○ Other, please list

Other - Specify

[ ]

Q14 organs declined

14. In the last 5 years has your program declined pediatric organs due to any of the following logistical/surgical resource limit? (select all that apply)

- ☐ Lack of available retrieval staff
- ☐ Lack of OR personnel (i.e. RNs, etc.)
- ☐ Lack of OR space availability
- ☐ Transportation (i.e. transportation of organs, retrieval teams, or recipients)
- ☐ Financial restrictions
- ☐ Recipient location
- ☐ No recipients on the waitlist
- ☐ Other, please list
- ☐ Not applicable – we have not had to decline pediatric organs due to logistical/surgical resource limitations

Other - Specify

[ ]

Q15 coroner restrictions

15. Are you aware of any circumstances in which a family was unable to proceed with donation due to restrictions from the medical examiner/coroner?

◯ Yes

◯ No

15a if yes, data collected

Complete only when:
Q15 coroner restrictions: Yes

Do you collect data on donation prevented by ME/Coroner?

◯ Yes

◯ No

15b if yes, process to address restrictions

Complete only when:
Q15 coroner restrictions: Yes

Do you have a process for addressing coroner restrictions?

◯ Yes, please describe

◯ No

Comments

Q16 policies/procedures/guidelines

The following questions are related to policies, procedures, guidelines, education, and staffing within your ODO.

16. Does your program have any policies, procedures or guidelines related specifically to pediatric donation?

- ○ Yes
- ○ No

Q17 standard guidelines

17. Does your program use any standardized guidelines for pediatric donor management?

- ○ Yes
- ○ No

17a if yes

Complete only when:
Q17 standard guidelines: Yes

Please select which of the following you use (select all that apply)

- ☐ Local
- ☐ National

☐ International

☐ Other, please list

Comments

Q18 champion

18. Is there anyone in your program identified as a champion or liaison specifically for pediatric or neonatal donation?

○ Yes

○ No

18a if yes

Complete only when:
Q18 champion: Yes

Is any education/training is provided to the person in this position?

○ Yes, please describe below

○ No

Comments

Q19 committees

19. Do you have any committees within your program with a focus on pediatric/neonatal donation?

○ Yes

○ No

19a if yes, family involvement

Complete only when:
Q19 committees: Yes

Is there family involvement in these committees?

○ Yes

○ No

Q20 training

20. Do your donor coordinators receive any pediatric/neonatal focused education/training?

○ Yes

○ No

20a if yes, type of training

Complete only when:
Q20 training: Yes

Which types of education/training are provided? (select all that apply)

- ☐ Annual ed days
- ☐ Online modules
- ☐ Simulation
- ☐ Lunch and learns
- ☐ Rounds
- ☐ Informal
- ☐ Other, please list

Other - Specify

Q21 review donor potential

21. Do you review all pediatric or neonatal deaths for donor potential (i.e. potential donor audit, death audit)?

- ○ Yes, we review all pediatric and neonatal deaths for donor potential
- ○ Yes, we review all pediatric, but NOT neonatal deaths for donor potential
- ○ Yes, we review all neonatal, but NOT pediatric deaths for donor potential

No

21a if yes, review frequency

Complete only when:
Q21 review donor potential: Yes, we review all pediatric and neonatal deaths for donor potential; Yes, we review all pediatric, but NOT neonatal deaths for donor potential; Yes, we review all neonatal, but NOT pediatric deaths for donor potential

How frequently are these reviews completed?

- Ad hoc
- Annually
- Quarterly
- Monthly
- Other, please list

Other - Specify

21b if yes, data sharing

Complete only when:
Q21 review donor potential: Yes, we review all pediatric and neonatal deaths for donor potential; Yes, we review all pediatric, but NOT neonatal deaths for donor potential; Yes, we review all neonatal, but NOT pediatric deaths for donor potential

Is this data shared/reported outside of your unit?

- ◯ Yes, please describe how it is shared and which whom
- ◯ No

Comments

21c if yes, process

Complete only when:
Q21 review donor potential: Yes, we review all pediatric and neonatal deaths for donor potential; Yes, we review all pediatric, but NOT neonatal deaths for donor potential; Yes, we review all neonatal, but NOT pediatric deaths for donor potential

What is your process when missed donors are identified? (select all that apply)

- ☐ Specified safety report
- ☐ Mortality review
- ☐ Donor committee reviews
- ☐ Ad hoc conversations
- ☐ Other, please describe

Other - Specify

Q22 database

The following questions are related to data collection for your ODO._

22. Do you have your own donor and or transplant database within your program?

- ◯ Yes, we have both a donation and transplant database
- ◯ Yes, we have a donation database
- ◯ Yes, we have a transplant database
- ◯ No, we do not have our own database

Q23 reporting to registries

23. Does your program report to any local, national or international registries?

- ◯ Yes, please list
- ◯ No

Comments

Q24 pediatric specific data

24. Does your program collect any pediatric specific data?

- ◯ Yes
- ◯ No

Q25 waitlist deaths

25. Do you record information on death from the pediatric waitlist?

- ◯ Yes
- ◯ No

Q26 removal from waitlist

26. Do you record information on removal for deterioration/too sick from the pediatric waitlist?

- ◯ Yes

○ No

Q27 reasons for decline

27. Does your ODO record reasons for pediatric organ decline by transplant program?

○ Yes

○ No

27a if yes, reasons

Complete only when:
Q27 reasons for decline: Yes

Please select which of the following reasons you have collected for pediatric organ decline (select all applicable):

☐ Size/weight of patient

☐ Size/weight of organ

☐ Age of donor

☐ DCD donor

☐ HLA matching

☐ Organ function/quality

☐ Recipient considerations

☐ Exceptional Distribution

☐ Donor distance/ cold ischemic time

☐ ABO

☐ Other, please list

Other - Specify

Q28 not offering organs

28. Does your ODO document reasons for not offering pediatric organs?

○ Yes (please specify)

○ No

28a if yes, reasons

Complete only when:
Q28 not offering organs: Yes (please specify)

Please select which of the following reasons you have collected for pediatric organ decline (select all applicable)

- ☐ Size/weight of patient
- ☐ Size/weight of organ
- ☐ Age of donor
- ☐ DCD donor
- ☐ HLA matching
- ☐ Organ function/quality
- ☐ Recipient considerations
- ☐ Exceptional Distribution
- ☐ Donor distance/ cold ischemic time
- ☐ ABO
- ☐ Other, please list

Other - Specify

Q29 recovered but not transplanted

29. Does your program collect data on pediatric organs recovered and not transplanted?

- ◯ Yes
- ◯ No

Q30 reporting publicly available

30. Does you ODO provide publicly available reports for any of the following? (select all that apply)

- ☐ Donation performance
- ☐ Missed donation opportunities
- ☐ Potential Donor Audit
- ☐ Transplant referral and waitlist performance
- ☐ Referral and waitlist performance
- ☐ Outcomes for recipients
- ☐ Wait list data
- ☐ Consent rate
- ☐ None of the above
- ☐ Other, Please list

Other - Specify

Q31 other barriers to donation

31. Are there any facilitators or barriers to pediatric/neonatal organ donation within your organization that we have not asked about?

○ Yes, please describe

○ No

Comments

Q32 contact info

Thank you for completing the survey.

In order to have a complete understanding of pediatric organ donation and transplantation practices in Canada, we would greatly appreciate if you could provide any of the following pediatric-specific documents for your program by emailing them to lee.james@blood.ca

- Pediatric specific absolute donor exclusion criteria for any donation and by organ.
- Documents used to track rational for decline of pediatric organs
- Data on rational for decline of pediatric organs
- Organ acceptance/refusal criteria
- Local allocation documents
- Registry reports
- Pediatric Data collection forms.
- Policies on recipient distance from transplant program
- Data on pediatric organs recovered and not transplanted
- Recipient management guidelines
- Data on short term post-transplant outcomes
- Data on long term post-transplant outcomes
- Guidelines for pediatric donor management

Thank you for completing the survey.

We ask that you provide contact information below, for a member of our team to be able to contact you should we require further clarification on one of your survey answers. This information will not be shared.

If you have any questions, please contact Laurie.lee@albertahealthservices.ca

Name

Email

Phone number
