# Supplementary material for: Variability in pediatric and neonatal organ offering, acceptance and utilization: a survey of Canadian pediatric transplant programs and organ donation organizations
Source: Front Transplant. 2024 Sep 27;3:1458563. doi: 10.3389/frtra.2024.1458563 (PMC11466726; doi:10.3389/frtra.2024.1458563)

Supplemental Figure 1 : Methods of communication used by ODOs for offering organs locally and nationally

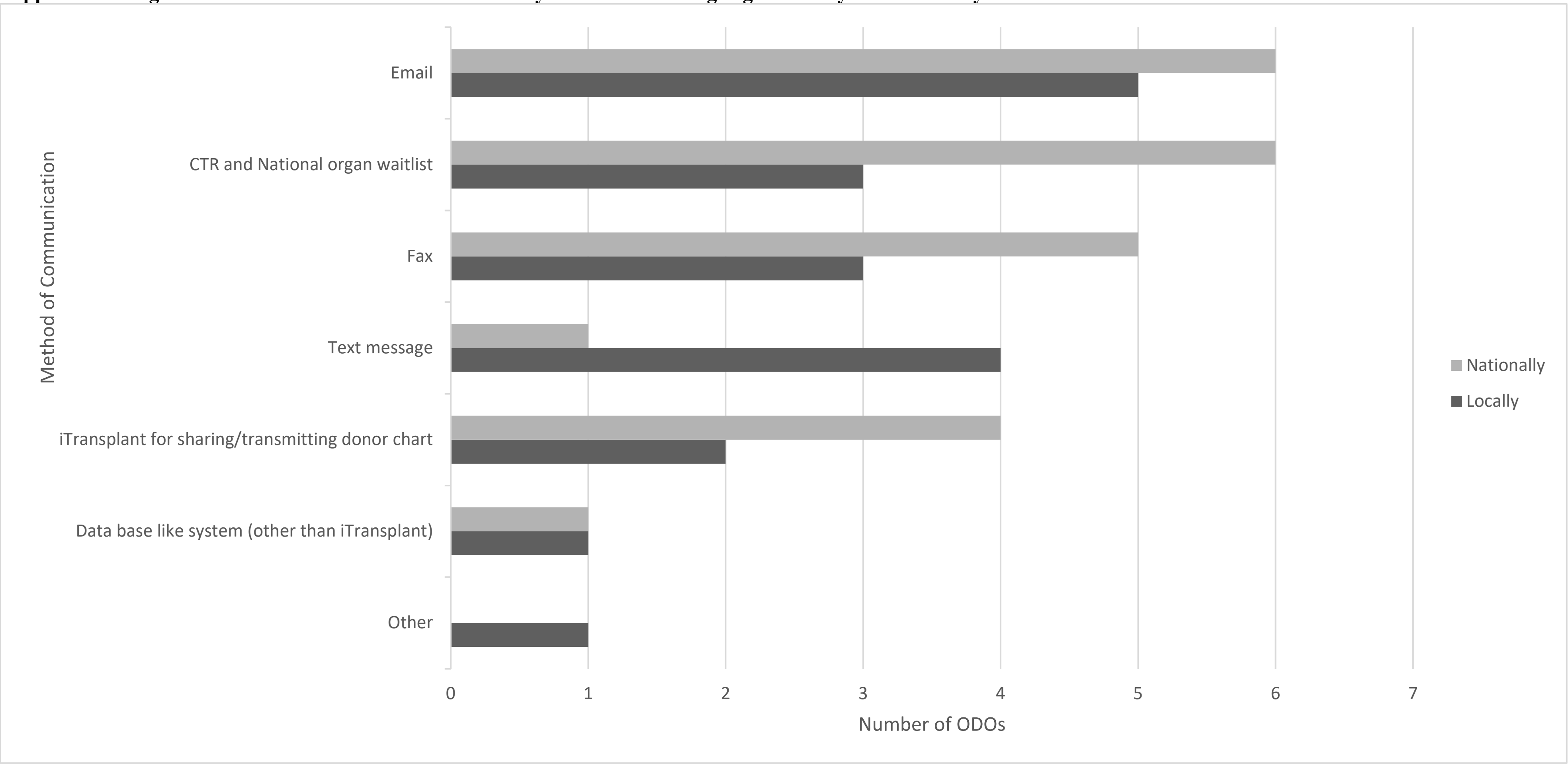

Supplemental Figure 2: Factors that inform organ allocation practices in Canadian pediatric transplant programs

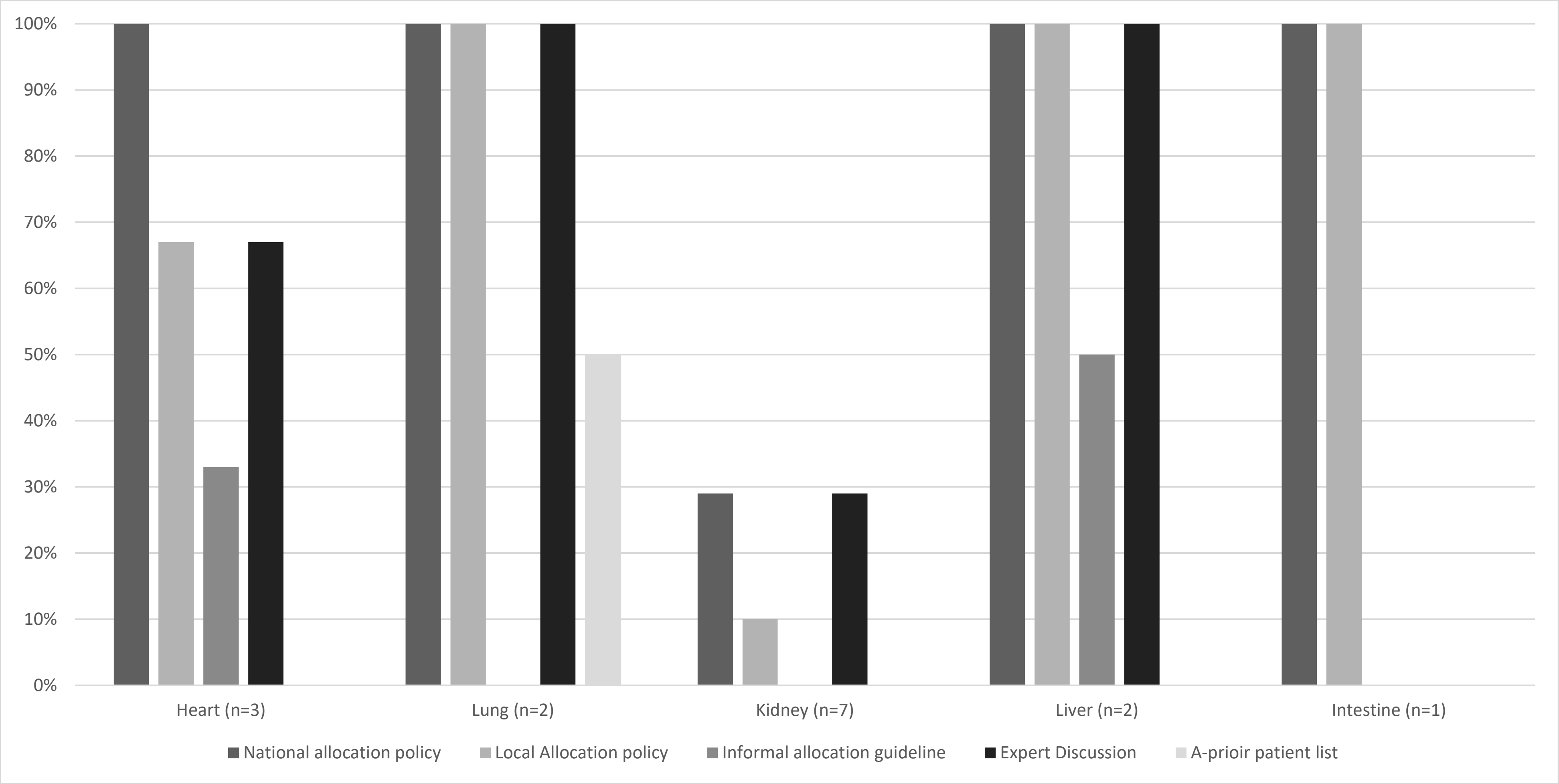

Supplement: Supplementary Data Sheet 3 — Supplementary Figures 1, 2. [file Datasheet3.pdf]
